# PARP9 and PARP14 cross-regulate macrophage activation via STAT1 ADP-ribosylation

**DOI:** 10.1038/ncomms12849

**Published:** 2016-10-31

**Authors:** Hiroshi Iwata, Claudia Goettsch, Amitabh Sharma, Piero Ricchiuto, Wilson Wen Bin Goh, Arda Halu, Iwao Yamada, Hideo Yoshida, Takuya Hara, Mei Wei, Noriyuki Inoue, Daiju Fukuda, Alexander Mojcher, Peter C. Mattson, Albert-László Barabási, Mark Boothby, Elena Aikawa, Sasha A. Singh, Masanori Aikawa

**Affiliations:** 1 Center for Interdisciplinary Cardiovascular Sciences, Cardiovascular Division, Brigham and Women's Hospital, Harvard Medical School, Boston, Massachusetts 02115, USA; 2 Channing Division of Network Medicine, Brigham and Women's Hospital, Harvard Medical School, Boston, Massachusetts 02115, USA; 3 Department of Physics, Center for Complex Network Research, Northeastern University, Boston, Massachusetts 02115, USA; 4 Department of Microbiology and Immunology, Vanderbilt University School of Medicine, Nashville, Tennessee 37232, USA; 5 Center for Excellence in Vascular Biology, Cardiovascular Division, Brigham and Women's Hospital, Harvard Medical School, Boston, Massachusetts 02115, USA

## Abstract

Despite the global impact of macrophage activation in vascular disease, the underlying mechanisms remain obscure. Here we show, with global proteomic analysis of macrophage cell lines treated with either IFNγ or IL-4, that PARP9 and PARP14 regulate macrophage activation. In primary macrophages, PARP9 and PARP14 have opposing roles in macrophage activation. PARP14 silencing induces pro-inflammatory genes and STAT1 phosphorylation in M(IFNγ) cells, whereas it suppresses anti-inflammatory gene expression and STAT6 phosphorylation in M(IL-4) cells. PARP9 silencing suppresses pro-inflammatory genes and STAT1 phosphorylation in M(IFNγ) cells. PARP14 induces ADP-ribosylation of STAT1, which is suppressed by PARP9. Mutations at these ADP-ribosylation sites lead to increased phosphorylation. Network analysis links PARP9–PARP14 with human coronary artery disease. PARP14 deficiency in haematopoietic cells accelerates the development and inflammatory burden of acute and chronic arterial lesions in mice. These findings suggest that PARP9 and PARP14 cross-regulate macrophage activation.

## Abstract (web summary)

Signalling pathways that mediate macrophage activation in disease are poorly understood. Here the authors show that inhibition of PARP9 and/or activation of PARP14 may attenuate macrophage-mediated vascular diseases, and also provide new insight into the development of effective therapies for other inflammatory disorders.

## Graphical abstract

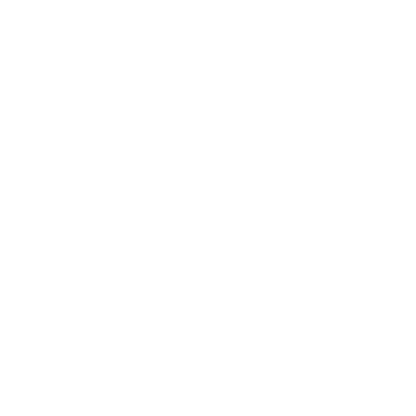

Despite medical advances, the global burden of ischaemic heart disease is increasing^1^,^2^. Pro-inflammatory macrophage activation plays key roles in the pathogenesis of many disorders, including arterial disease^3^,^4^,^5^,^6^,^7^,^8^,^9^,^10^. Some pathways associated with macrophage activation may contribute to the shared mechanisms of inflammatory diseases, as demonstrated previously^11^,^12^. Despite potent therapies such as cholesterol-lowering by statins, substantial residual cardiovascular risk remains^7^,^13^,^14^, which drives the active search for novel solutions against pro-inflammatory macrophage activation. Dissecting complex and intertwined mechanisms for macrophage activation requires well-defined mechanistic models. The evidence suggests that distinct types of macrophage activation are functionally different in disease pathogenesis, a classification that has helped to assess the heterogeneity of macrophages^15^,^16^,^17^,^18^,^19^,^20^,^21^,^22^. For instance, pro-inflammatory and anti-inflammatory phenotypes can oppose one another, develop in response to distinct cytokines, differ in the activating stimuli and produce different cytokines. A recently proposed nomenclature suggests that each macrophage subpopulation can be called based on a specific stimulator, for example, M(IFNγ), M(LPS), M(IL-4), M(IL-10)^21^. This established paradigm demonstrates clear relationships between classical stimuli and their respective responses—interferon gamma (IFNγ) for pro-inflammatory activation in settings such as atherosclerotic vascular disease and interleukin (IL)-4 for activation that can counter that of M(IFNγ) or M(LPS) macrophages. Hence, we used this paradigm as a starting point to explore novel regulators through global proteomics.

Proteomics screening and bioinformatics in mouse and human data sets found that poly ADP-ribose polymerase 14 (PARP14), also known as ADP-ribosyltransferase diphtheria toxin-like 8 (ARTD8), and PARP9/ARTD9 both increased in M(IFNγ) and decreased in M(IL-4) cells. The network analysis associated these PARP family members with human arterial disease. Sequence similarity to the PARP catalytic domain, which transfers ADP-ribose moieties from NAD to protein acceptors, characterizes the PARP family proteins^23^. The best-characterized member, PARP1/ARTD1, represents poly-ADP-ribosylation enzymes, which processively catalyse long and branching polymers of ADP-ribose additions starting from an initial post-translational modification, commonly of glutamate. Recent evidence also validates proteins that execute mono-ADP-ribosylation as having various functions^24^. PARP14/ARTD8 is an intracellular mono-ADP-ribosyltransferase. Previous reports indicated that PARP14 enhances IL-4-induced gene expression by interacting with the cytokine-induced signal transducer and activator of transcription 6 (STAT6) in B and T cells, thereby functioning as a transcriptional co-activator^25^,^26^ that may mediate this effect. A recent study reported that PARP14 regulates the stability of tissue factor mRNA in M(LPS) in mouse^27^. Less information exists regarding the molecular function of PARP9/ARTD9. Although PARP9 appears to lack catalytic activity^28^, it increases IFNγ-STAT1 signalling in B-cell lymphoma^29^.

This study employed a multidisciplinary approach, including proteomics, systems biology and cell and molecular biology to explore new mechanisms for modulating the functional profile elicited after macrophage activation. Mouse and human cell lines as well as primary macrophages were used for complementary analyses of PARP14-deficient mouse and human tissues. Ultimately, the analyses led to evidence that expression of PARP14 in haematopoietic cells restrains vascular inflammation in mouse models, which are not solely regulated by either IFNγ or IL-4. Our findings suggest a novel mechanism for regulating the balance of macrophage phenotypes in vascular disease, and potentially other disorders in which macrophage activation has an impact on outcomes.

## Results

### Proteomics screening for regulators of macrophage activation

We used the tandem mass tagging (TMT) quantitative proteomics to identify regulators of pro-inflammatory and non/anti-inflammatory activation in mouse RAW264.7 and human THP-1 macrophage cell lines (Supplementary Fig. 1a–c). In this paradigm of macrophage heterogeneity, IFNγ and IL-4 promote distinctive subpopulations^15^,^16^,^17^,^18^,^19^,^20^,^21^,^22^. A pilot TMT proteomic study (Supplementary Figs 1d and 2) analysed the changes in the proteomes at 0, 12 and 24 h, and observed the expected increase and decrease in STAT1 in M(IFNγ) and M(IL-4) cells, respectively, as determined by hierarchical cluster analysis (Supplementary Fig. 2). Within this pilot study, we first noted that PARP14 co-clustered with STAT1 in the M(IFNγ) and M(IL-4) data (Supplementary Fig. 2). To ascertain whether any changes in the M(IFNγ) and M(IL-4) proteomes were not because of cell culture conditions, we performed a second, more in-depth study that included an unstimulated macrophage control for both RAW264.7 and THP-1 experiments, and extended the stimulation period to up to 72 h, sampling six time points for a more detailed time-resolved proteomic study (Supplementary Fig. 1d).

**Figure 1:**
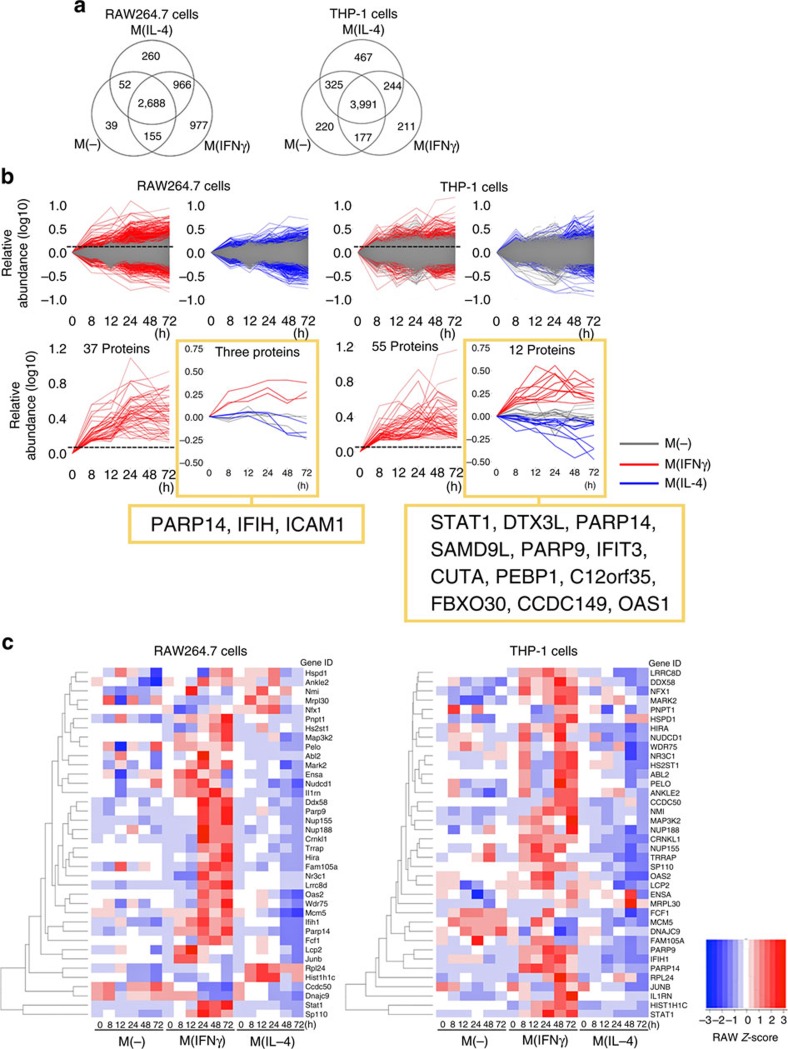
Bioinformatics to identify candidate regulators of macrophage activation. (**a**) Venn diagrams showing the distribution of quantified proteins from mouse RAW264.7 and human THP-1 cells in unstimulated control, IFNγ-stimulated and IL-4-stimulated macrophages: M(-), M(IFNγ) and M(IL-4), respectively. (**b**) Data set-filtering strategy. Upper panels: superimposition of the 0-h-normalized protein abundance profiles for M(-) (grey traces) versus M(IFNγ) (red traces) or M(IL-4) (blue traces) data sets in RAW264.7 and THP-1 cells. Lower panels: extracted protein profiles of interest generated by data filtering. Red traces only graphs: extracted profiles of proteins whose abundances exceed the M(IFNγ) threshold (+0.13, maximum protein abundance in unstimulated control at 8 h, dashed line). From these M(IFNγ)-filtered traces, those that decreased in IL-4 stimulation when compared with their unstimulated control are plotted to the right for RAW264.7 and THP-1 cells, respectively. (**c**) Hierarchical clustering of 38 proteins from that were identified in both RAW264.7 and THP-1 data sets. Each row corresponds to a protein gene ID.

In this latter proteomic study, we quantified 5,137 and 5,635 proteins in RAW264.7 and THP-1 cells, respectively, across the three conditions: unstimulated control, IFNγ-stimulated macrophages and IL-4-stimulated macrophages—M(-), M(IFNγ) and M(IL-4), respectively (Fig. 1a and Supplementary Table 1). An overview of the protein intensities across the three conditions revealed that the magnitude of protein abundance levels for each of the IFNγ and IL-4-stimulated RAW264.7 and THP-1 cells were generally higher than those of unstimulated cells (Fig. 1b), indicating that each stimulation promoted changes in protein abundance beyond those due to cell culture conditions alone.

To pursue one class of potential upstream regulators based on protein abundances, we used the following criteria: proteins exhibited (1) an early increase in M(IFNγ) (within 24 h) followed by sustained levels until the later time points (up to 72 h), (2) a decrease in abundance in M(IL-4) and (3) no significant change in M(-). We employed two distinct informatics methods to explore proteins with such behaviours: data filtering (Method 1) and model-based clustering (Method 2; Supplementary Fig. 3). In Method 1, the M(-) data set permitted a subtraction of the background signal at all time points and thus facilitated data filtering (Fig. 1b upper panels and Supplementary Fig. 3b). Three proteins in the data set from mouse RAW264.7 cells and 12 proteins in human THP-1 cells (Fig. 1b lower panels) met the three criteria above. PARP14 emerged from both data sets, and PARP9 appeared in the THP-1 data set (Fig. 1b lower panels). In parallel, Method 2 (Supplementary Fig. 3c) produced 15 and 20 clusters for the RAW264.7 and THP-1 data sets, respectively (Supplementary Fig. 4a). Clusters mined for proteins whose abundances increased in M(IFNγ) but decreased in M(IL-4) with respect to M(-) revealed 490 proteins in the RAW264.7 data set and 414 proteins in THP-1 fulfilling these criteria (Supplementary Fig. 4b and Supplementary Tables 2 and 3). The proteins were short-listed to those with similar time-resolved changes in both in human and mouse data sets, resulting in 38 candidate proteins (Fig. 1c). Both RAW264.7 and THP-1 data sets identified PARP9 and PARP14 (Supplementary Fig. 4a). Collectively, while Method 2 identified PARP9 and PARP14 in RAW264.7 and THP-1 cells, PARP14 was the only common protein that both Methods 1 and 2 identified among over 5,000 proteins in mouse and human data sets.

### The PARP9 and PARP14 network and coronary artery disease

To understand the influence of PARP9 and PARP14 in a global interaction network (‘interactome') and predict their potential clinical impact in disease mechanisms, we applied a network-based analysis (Supplementary Fig. 5a). Increasing evidence suggests that disease genes are not distributed randomly on the interactome but work together in similar biological modules or pathways^30^,^31^. Moreover, gene products (for example, proteins) linked to the same phenotype likely interact with each other and cluster in the same network neighbourhood^31^. We thus postulated that, if PARP9 or PARP14 influences the network neighbourhood of a disease, its immediate neighbours should be close to a disease module compared with random expectation^30^,^31^. Using the random-walk method, we defined a set of genes as a human disease module for each of the cardiovascular and metabolic diseases, and IFNγ-related diseases (see Methods). We then measured the average shortest distance of the immediate neighbours of the PARP9–PARP14 network to each disease module. An interactome mainly describes a set of physical or functional associations between proteins and does not provide cell or tissue specificity. In our study, we thus examined the genes of immediate neighbours reported in macrophages in the public databases.

**Figure 2:**
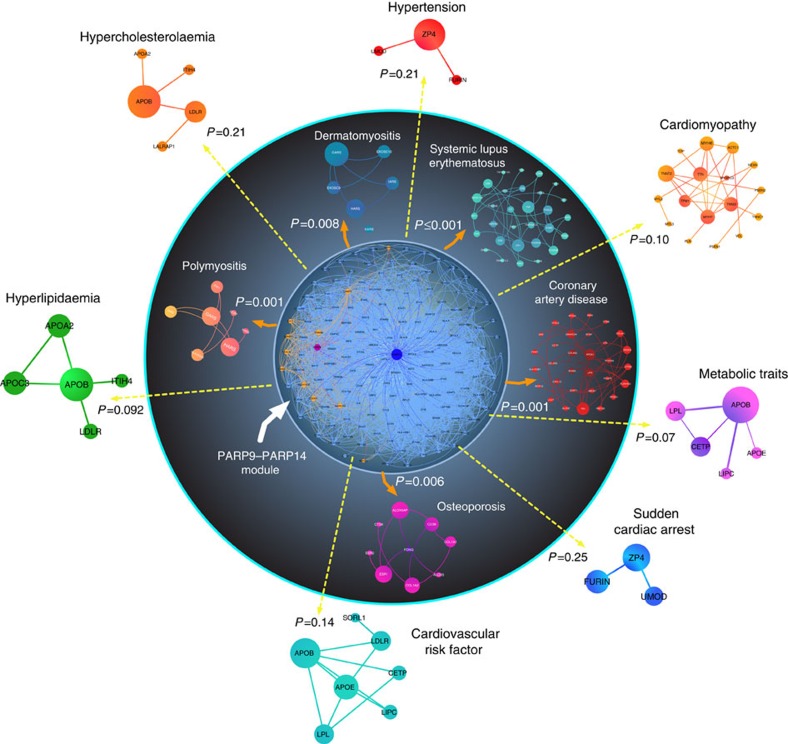
Network analysis links PARP9–PARP14 with coronary artery disease. The PARP14 (blue)–PARP9 (purple) module consists of the first neighbours of each protein (light blue and orange nodes, respectively). The significance of closeness of the PARP9–PARP14 first neighbours in the interactome (PARP9–PARP14 module) and disease modules compared with random expectation is indicated by *P* values. The random expectation was same size-connected components of PARP9–PARP14 module and a disease module drawn randomly from the network. Closeness between PARP9–PARP14 modules and other diseases such as cardiovascular, metabolic and IFNγ-related diseases was evaluated in the network. The inner circle contains significantly close disease modules.

We included IFNγ-related autoimmune diseases as positive controls, as we used this cytokine to promote pro-inflammatory macrophage activation. The PARP9–PARP14 network is significantly close to systemic lupus erythematosus, dermatomyositis and polymyositis, as expected (Fig. 2). The PARP9–PARP14 network had significantly greater proximity to the human coronary artery disease gene module compared with other cardiovascular and metabolic diseases (Fig. 2 and Supplementary Fig. 5b). The analysis also linked PARP9–PARP14 with osteoporosis. Moreover, we quantified the closeness of PARP9–PARP14 to autoimmune diseases, coronary artery disease and other diseases in the form of the distribution of shortest distances (Supplementary Fig. 5c) and demonstrated a clear separation between the distribution of diseases inside and outside the circle (Fig. 2), indicating an enrichment of shorter distances (*d*=1 and 2) for IFNγ-related autoimmune diseases (yellow) and coronary artery disease and osteoporosis (blue), compared with other cardiovascular and metabolic diseases (red; Supplementary Fig. 5c). These results may indicate the potential impact of PARP9 and/or PARP14 on the pathogenesis of arterial disease or the onset of its clinical complications.

### *In vitro* validation in cultured macrophages

**Figure 3:**
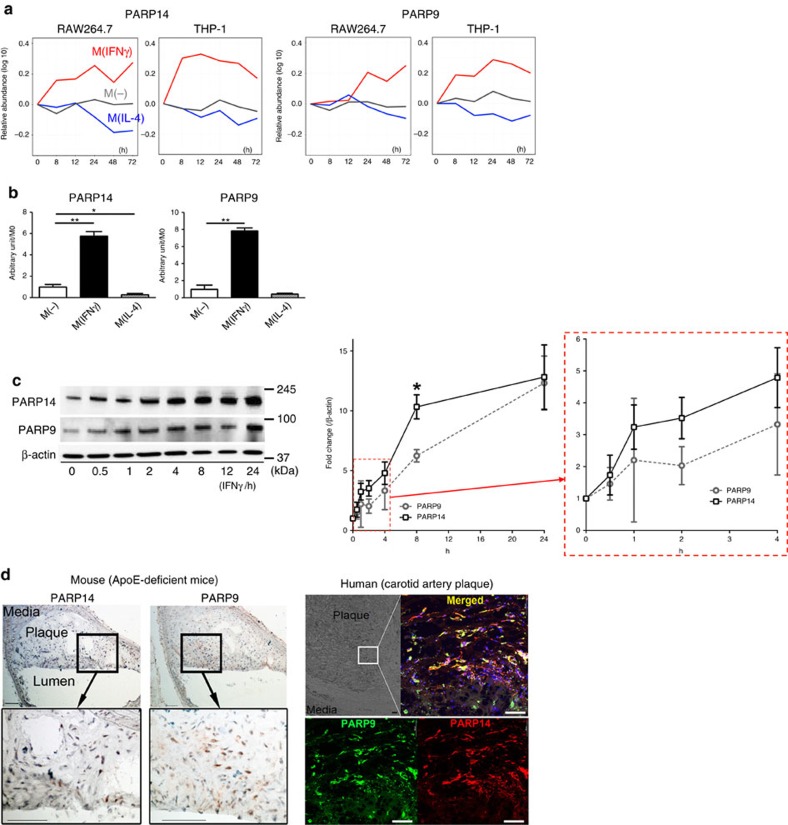
PARP9 and PARP14 expression *in vitro* and *in vivo*. (**a**) TMT-derived 0-h-normalized protein abundance profiles for PARP9 and PARP14 from mouse RAW264.7 and human THP-1 M(IFNγ) and M(IL-4) data sets. (**b**) PARP9 and PARP14 gene expression at 24 h after stimulation (*n*=3). (**c**) PARP9 and PARP14 protein expression visualized by western blot. The time course in the relative protein abundances of PARP9 and PARP14 normalized to β-actin were quantified (graph, *n*=3). *P<0.05 and **P<0.01, respectively, by Student's *t*-test. Error bars indicate s.d. (**d**) Representative images of PARP9 and PARP14 expression in atherosclerotic plaques from the aorta of an *Apoe*^−/−^ mouse (*n*=3) fed a high-fat diet and from the carotid artery of a human (*n*=5). Scale bars, 100 μm.

qPCR and western blot analysis validated the proteomic screening data on PARP9 and PARP14. Consistent with the proteomics data (Fig. 3a and Supplementary Fig. 4a), mRNA and protein levels of PARP9 and PARP14 increased with IFNγ and decreased by IL-4 (Fig. 3b and Supplementary Fig. 6). At protein levels, PARP14 increased before PARP9 in response to IFNγ (Fig. 3c).

### PARP9 and PARP14 expression in plaque macrophages

According to the network analysis that linked PARP9 and PARP14 with arterial disease, we performed immunohistochemistry in arterial lesions. Mouse (Fig. 3d, left) and human (Fig. 3d, right) atherosclerotic lesions exhibited PARP9 and PARP14 proteins, while they were less abundant in human carotid arteries with no apparent atherosclerotic changes (Supplementary Fig. 7a). Immunohistochemistry localized PARP9 and PARP14 expression in the majority of macrophages (CD68) of human atherosclerotic plaques, while few if any smooth muscle cells (SMα-actin) and endothelial cells (CD31) stained positively for these PARPs (Supplementary Fig. 7b). These results suggest that macrophages are a major source of PARP9 and PARP14 in human atherosclerotic lesions.

### Pro-inflammatory PARP9 and anti-inflammatory PARP14

A series of subsequent experiments examined whether PARP9 or PARP14 plays a causal role in macrophage activation. Expression levels of gene products typical of the M(IFNγ) phenotype (for example, IL-1β) and in the M(IL-4) phenotype (for example, MRC1) gauged the downstream effects of PARP silencing using small interfering RNA (siRNA) first in macrophage-like cell lines RAW264.7 and THP-1 since the original screening was performed in these cells, and then in human and mouse primary macrophages.

In mouse RAW264.7 cells, PARP14 silencing enhanced the induction of tumour necrosis factor alpha (TNFα) and inducible nitric oxide synthase (iNOS) by IFNγ, and suppressed the response of MRC1 to IL-4 (Supplementary Fig. 8a). In human THP-1 cells, PARP14 silencing also increased TNFα and IL-1β mRNA induction in response to IFNγ and decreased MRC1 induction by IL-4 (Supplementary Fig. 8b). Increased levels of TNFα and IL-1β proteins in the supernatant of IFNγ-treated THP-1 cells with PARP14 silencing (Supplementary Fig. 8c) supported these data. Silencing of PARP9 suppressed the induction of TNFα, IL-1β and CCL2/MCP-1 mRNA under IFNγ stimulation in THP-1 cells, while MRC1 showed no significant change in IL-4 (Supplementary Fig. 8d).

**Figure 4:**
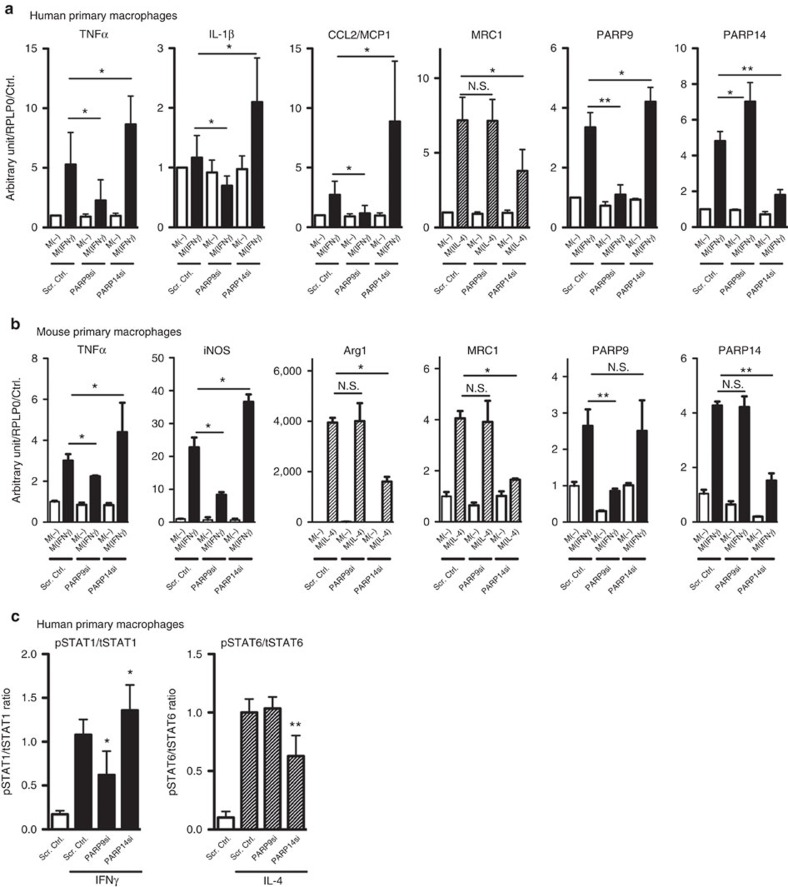
The molecular functions of PARP9 and PARP14 in macrophages *in vitro*. (**a**) The consequences of PARP9 and PARP14 silencing on IFNγ stimulated (TNFα, IL-1β and CCL2/MCP-1) and IL-4 stimulated (MRC1) gene expression in human primary macrophages (*n*=8). (**b**) The consequences of PARP9 and PARP14 silencing on IFNγ stimulation (TNFα and iNOS) and IL-4 stimulation (Arg1 and MRC1) gene expression in mouse bone marrow-derived macrophages (*n*=3). (**c**) The ratio of phosphorylated STAT1 and STAT6 protein levels to total STAT1 and STAT6 (pSTAT1/tSTAT1 ratio and pSTAT6/tSTAT6 ratio) in human primary macrophages (*n*=6 and *n*=5, respectively) of the PARP9 and PARP14 silencing experiments. * *P*<0.05 and **P<0.01, respectively, by Student's *t*-test. Error bars indicate s.d.

To obtain unambiguous evidence for the role of PARP9 and PARP14 in macrophage activation, we extended the *in vitro* validation studies to mouse and human primary macrophages. In human primary macrophages derived from CD14+ peripheral blood mononuclear cells (PBMCs), PARP9 silencing suppressed the expression of TNFα, IL-1β and CCL2/MCP-1 in IFNγ-treated macrophages, but exerted no significant effects on MRC1 induction by IL-4 (Fig. 4a). In contrast, PARP14 silencing accelerated the induction of TNFα, IL-1β and CCL2/MCP-1 by IFNγ, and suppressed MRC1 in IL-4-treated macrophages (Fig. 4a). In mouse bone marrow-derived macrophages (BMDMs), silencing PARP9 and PARP14 exerted the effects similar to those in human primary macrophages (Fig. 4b). Overall, siRNA experiments provided consistent results in macrophage-like cell lines and primary macrophages. Neither PARP14 nor PARP9 showed significant effects on viability, proliferation or apoptosis of mouse primary macrophages (Supplementary Fig. 8e). In addition, enforced expression of PARP14 in THP-1 cells suppressed the induction of TNFα, iNOS, TLR2 and TLR4 in M(IFNγ) (Supplementary Fig. 8f,g). Collectively, these findings indicate that PARP14 suppresses IFNγ-induced responses and augments IL-4-responses in macrophages. In contrast, PARP9 promotes responses to IFNγ.

### PARP9 and PARP14 regulate STAT1 and STAT6 activation

Although expression patterns of PARP14 and PARP9 in M(IFNγ) and M(IL-4) were comparable, their respective siRNA experiments yielded opposing results, suggesting the involvement of distinct signalling mechanisms. IFNγ signalling involves activation (phosphorylation) of pro-inflammatory STAT1, while the IL-4 pathway uses anti-inflammatory STAT6 phosphorylation^32^,^33^. Immunofluorescence staining demonstrated enhanced intracellular colocalization of PARP14 and STAT1 in IFNγ-treated THP-1 cells compared with unstimulated cells (Supplementary Fig. 9). Moreover, in human primary macrophages derived from CD14+ PBMCs, PARP14 silencing accelerated IFNγ-induced STAT1 phosphorylation and suppressed IL-4-promoted STAT6 phosphorylation (Fig. 4c). PARP14 may suppress pro-inflammatory macrophage activation by modulating the IFNγ–STAT1 axis, and promote the anti-inflammatory IL-4-STAT6 pathway. In contrast, PARP9 silencing decreased STAT1 phosphorylation in IFNγ-treated human macrophages (Fig. 4c), indicating that PARP9 may activate IFNγ–STAT1 signalling and induce pro-inflammatory activation. In addition, siRNA experiments in THP-1 cells that we used for proteomic screening produced similar results on the role of PARP9 and PARP14 on the STAT1 and STAT6 pathways (Supplementary Fig 10a–c). Evidence suggests the participation of STAT3 in immune responses in various contexts^34^. Neither PARP14 nor PARP9 silencing, however, exerted significant effects on STAT3 phosphorylation (Supplementary Fig. 10d,e).

### PARP9 and PARP14 interact with each other

**Figure 5:**
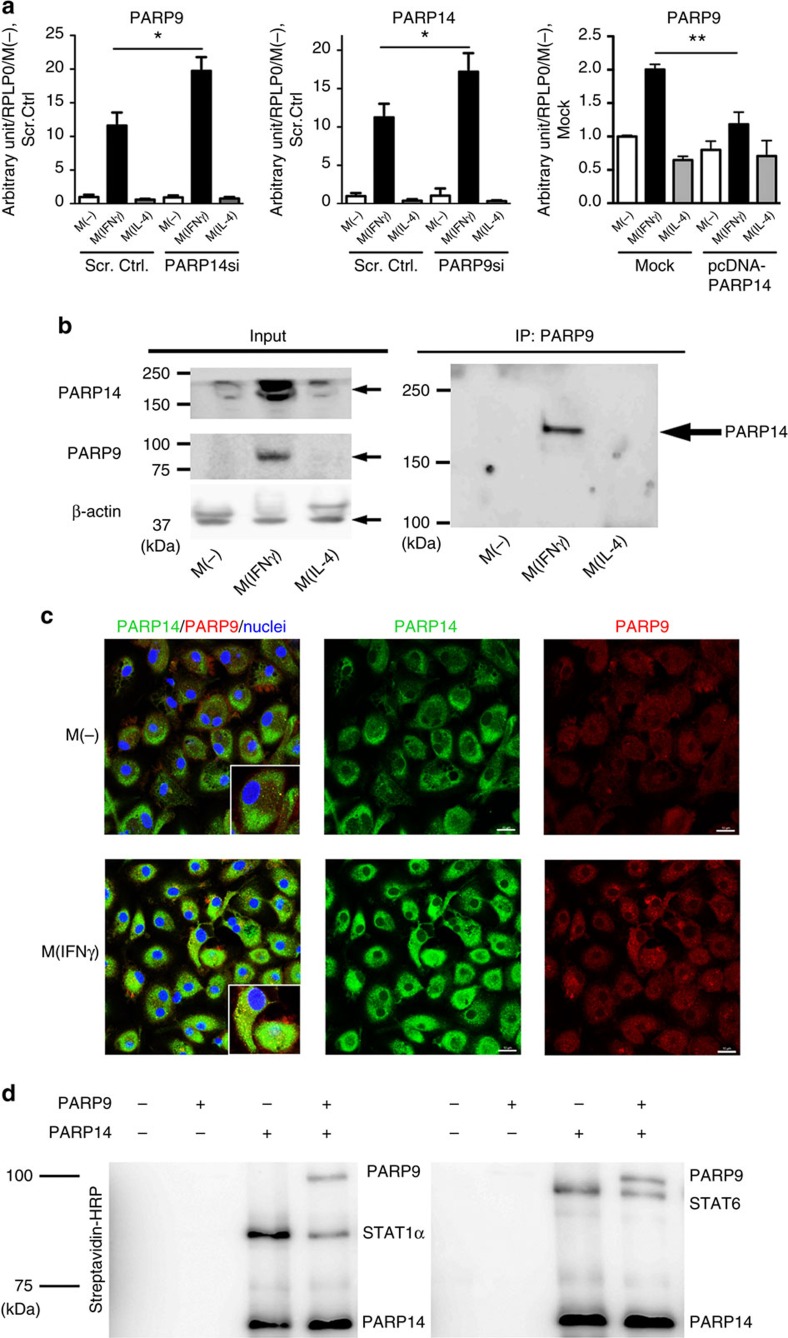
Potential interaction of PARP9 and PARP14. (**a**) PARP14 silencing and enforced expression significantly affected *PARP9* gene expression in IFNγ-stimulated THP-1 cells (*n*=3). PARP9 silencing increased PARP14 gene expression (*n*=3). (**b**) Co-IP assay revealed a complex between PARP9 and PARP14. (**c**) Intracellular colocalization of PARP9 and PARP14 in the cytosol in M(-) and M(IFNγ). (**d**) PARP9 inhibits ADP-ribosylation of STAT1α and STAT6 by PARP14 (protein ribosylation assay). PARP14 auto-ribosylation is also indicated. *P<0.05 and **P<0.01, respectively, by Student's *t*-test. Error bars indicate s.d.

**Figure 6:**
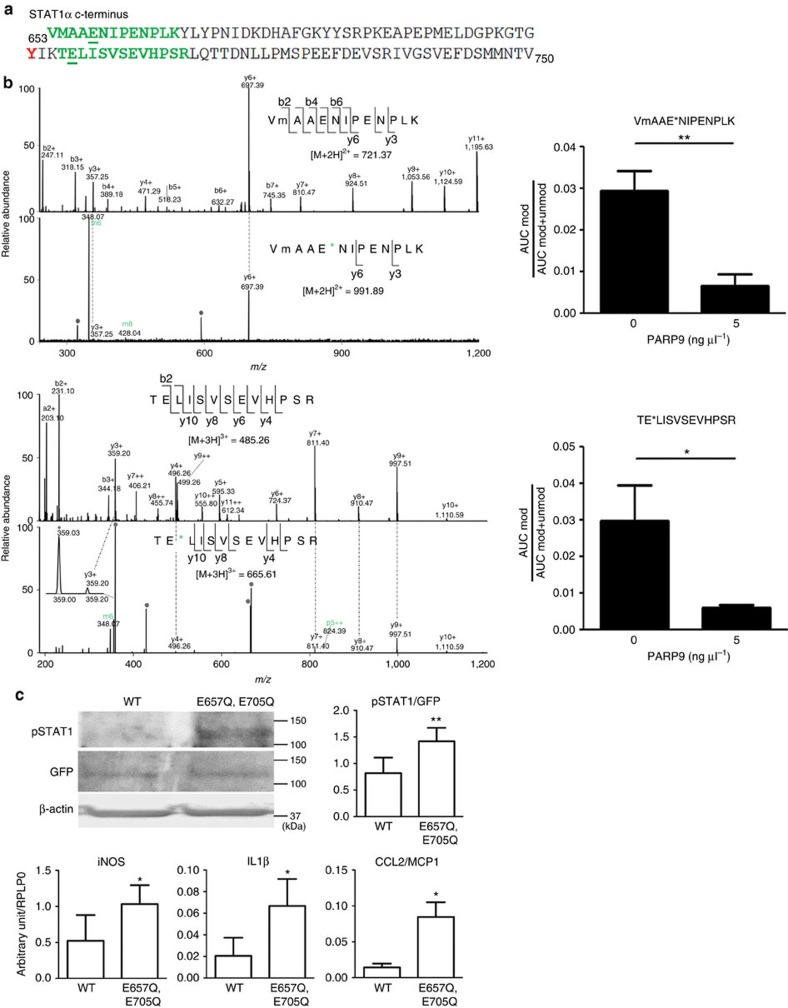
Identification of PARP14-induced ribosylation sites in STAT1. (**a**) The amino-acid sequence of human STAT1α C terminus. Green amino acids indicate ribosylated peptides; confirmed ribosylation sites are underlined. STAT1 is phosphorylated at indicated tyrosine (red). (**b**; Left panels) MS/MS spectra for the mono-ADP-ribosylated peptides and corresponding unmodified forms. ADP-ribose fragments are annotated in green. *, ribosylation site; m, oxidized methionine. The grey circles indicate background or undetermined ions. (Right panels) MS1-based quantification of PARP9 inhibition of PARP14-mediated STAT1α ribosylation at E657 (upper panel) and E705 (lower panel), respectively. (**c**) Effects of mutated amino acids at E657 and E705 in STAT1 (ribosylation sites for PARP14) on its Tyr701 phosphorylation and pro-inflammatory gene expression in mouse bone marrow-derived macrophages (*n*=4). *P<0.05 and **P<0.01, respectively, by Student's *t*-test. Error bars indicate s.d.

The results demonstrated above engendered the hypothesis of regulatory interplay between PARP9 and PARP14. Indeed, PARP14 silencing increased PARP9 mRNA expression in IFNγ-treated human primary macrophages (Fig. 4a) and THP-1 cells (Fig. 5a), while PARP9 silencing increased PARP14 mRNA in these cell types (Figs 4a and 5a). In contrast, enforced expression of PARP14 decreased PARP9 mRNA expression in IFNγ-treated THP-1 cells (Fig. 5a). Previous reports showed that, in B lymphocytes, IL-4 promotes catalytic activity of PARP14, leading to ADP-ribosylation of HDAC2, HDAC3 and p100 (a precursor of p52 that encodes the NF-κB2 protein), and activation of STAT6, thereby inducing its binding to IL-4-responsive gene promoters^25^,^26^,^35^,^36^. Co-immunoprecipitation (IP) revealed a PARP9/PARP14 complex (Fig. 5b) and immunofluorescence demonstrated the enhanced colocalization of PARP9 and PARP14 in THP-1 cells by IFNγ stimulation (Fig. 5c), suggesting that these two molecules interact in macrophages. Recombinant PARP14 protein induced ADP-ribosylation of PARP14 itself, PARP9, STAT1α and STAT6 (Fig. 5d). PARP9 did not promote ADP-ribosylation of either STAT1α or STAT6, which supports a previous report showing that PARP9 lacks catalytic activity^28^. Interestingly, PARP9 suppressed PARP14-induced ADP-ribosylation of STAT1α and STAT6 (Fig. 5d). While the majority of other PARP family members such as PARP1 are poly-ADP-ribosylation enzymes, PARP14 is a mono-ADP-ribosyltransferase^24^. Mass spectrometric analysis determined that Glu657 and Glu705 of STAT1α were mono-ADP-ribosylated by PARP14 (Fig. 6a,b). Glu657 and Glu705 neighbour Tyr701, a functionally critical phosphorylation site of STAT1α (Fig. 6a). Although we could not verify the precise ADP-ribosylation site in the STAT6 peptide, the conserved Glu makes it a plausible candidate (Supplementary Fig. 11). Mass spectrometry further revealed that recombinant PARP9 inhibited PARP14-induced mono-ADP-ribosylation at Glu657 and Glu705 of STAT1α (Fig. 6b, right panels).

The introduction of mutations at Glu657 and Glu705 to prevent ADP-ribosylation helped to investigate the functional relevance of these two sites within STAT1α. Overexpression experiments in mouse BMDMs revealed that mutations at Glu657 and Glu705 enhanced Tyr701 phosphorylation of STAT1α in IFNγ-treated cells as compared with wild-type STAT1α (Fig. 6c). Using the same mutant STAT1α, we have observed a similar response of STAT1α phosphorylation in HEK293 cells (Supplementary Fig. 12a,b). As a functional consequence, mutant STAT1α in mouse BMDMs increased expression of pro-inflammatory iNOS, IL-1β and CCL2/MCP-1 (Fig. 6c). Collectively, these data indicate that PARP14-mediated ADP-ribosylation of Glu657 and Glu705 may control Try701 phosphorylation of STAT1α, a potential mechanism for the interplay between ADP-ribosylation and phosphorylation in macrophage activation.

### PARP14 deletion enhances acute arterial lesion development

**Figure 7:**
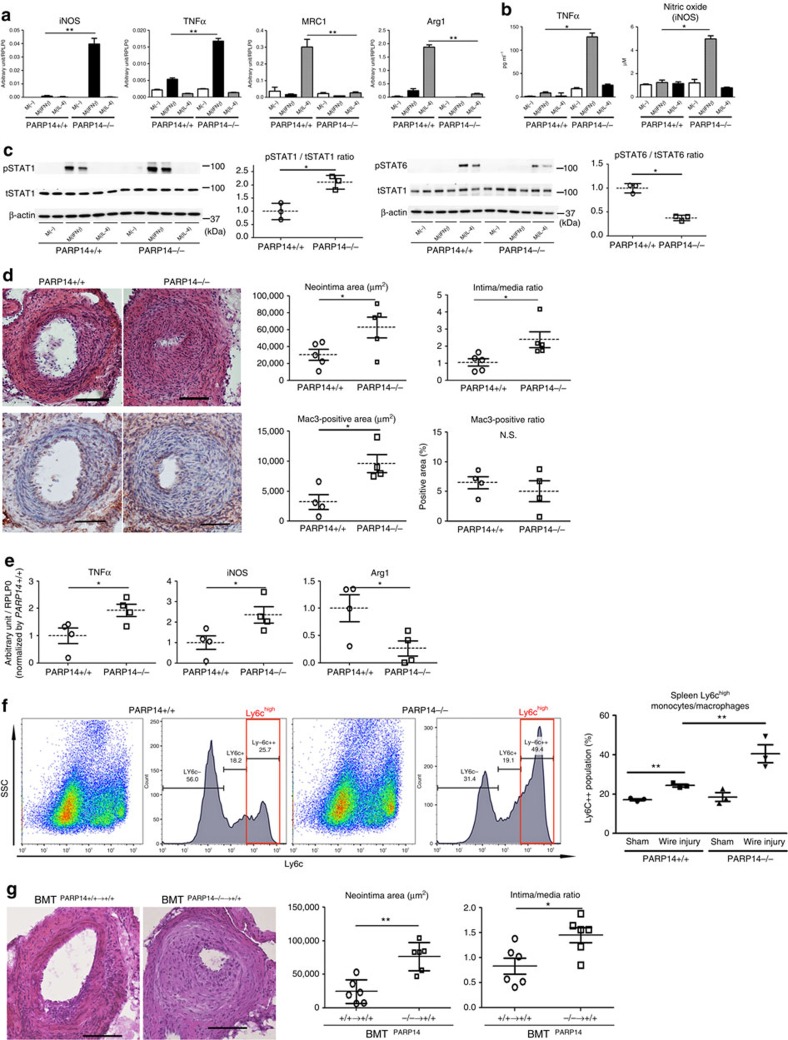
Role of haematopoietic PARP14 in acute arterial lesion formation in mice. (**a–c**) Cultured peritoneal macrophages derived from *PARP14*^−/−^ and *PARP14*^+/+^ mice. (**a**) IFNγ and IL-4 pathway gene expression profiles (*n*=3). (**b**) Secretion of inflammatory factors into culture media (*n*=3). (**c**) Western blot and corresponding densitometry quantification of phosphorylated STAT1 and STAT6. Each data point is the average of triplicate samples per donor (*n*=3). (**d**) Left: representative images of haematoxylin and eosin (H&E; top) and Mac3 (bottom) staining. Scale bars, 100 μm. Right: quantification of lesion formation in mechanically injured femoral arteries of *PARP14*^−/−^ and PARP^+/+^ mice. Mac3 staining represents macrophage accumulation (*n*=4–5). (**e**) LCM of the neointima followed by gene expression analysis (*n*=4). (**f**) Flow cytometry analysis of splenic CD11b+Ly6G− monocytes after induction of mechanically injured femoral arteries of *PARP14*^+/+^ and *PARP14*^−/−^ mice (*n*=3). (**g**) Representative H&E staining images and quantification of neointima formation in mechanically injured femoral arteries after bone marrow transplantation (BMT) *PARP14*^+/+→+/+^ and *PARP14*^−/−→+/+^ mice (*n*=6). Scale bars, 100 μm. * *P*<0.05 and **P<0.01, respectively, by Student's *t*-test. Error bars indicate s.d.

To provide *in vivo* evidence that PARP14 participates in arterial lesion formation and macrophage activation, we further used *PARP14*^−/−^ mice. Peritoneal macrophages from *PARP14*^−/−^ or *PARP14*^+/+^ mice enabled the examination of the macrophage phenotype. *PARP14*^−/−^ macrophages expressed higher mRNA and protein levels of iNOS and TNFα under IFNγ stimulation and lower levels of MRC1 and Arg1 mRNAs under IL-4 stimulation compared with *PARP14*^+/+^ cells (Fig. 7a,b). PARP14 deficiency also enhanced phosphorylation of STAT1 induced by IFNγ and decreased STAT6 phosphorylation in IL-4-treated peritoneal macrophages (Fig. 7c). BMDMs of *PARP14*^+/+^ and *PARP14*^−/−^ mice supported our results (Supplementary Fig. 13a). These results in *PARP14*^−/−^ macrophages are consistent with those of *in vitro* siRNA experiments.

Our network analysis closely linked the PARP9–PARP14 module with coronary artery disease (Fig. 2). To first examine whether PARP14 indeed plays a role in arterial diseases, we used two different models: (1) acute mechanical injury in femoral arteries of *PARP14*^−/−^ mice and (2) acute injury in mice that underwent bone marrow transplantation from *PARP14*^−/−^ mice. In a model of acute arterial responses due to wire-mediated mechanical injury, PARP14 deficiency enhanced neointima formation (Fig. 7d, top panels) and increased macrophage accumulation (Fig. 7d, bottom panels). Laser capture microdissection (LCM) of the intima of injured femoral arteries followed by real-time PCR demonstrated that PARP14 deficiency increased the levels of TNFα and iNOS mRNA and decreased Arg1 mRNA (Fig. 7e). The spleen is a reservoir of monocytes/macrophages that releases these cells in response to an event in remote organs^37^. Flow cytometry of splenic cells revealed that, in CD11b+Ly6G− monocytes/macrophages (Supplementary Fig. 13b), acute arterial injury increased the Ly6C^high^ population in *PARP14*^+/+^ mice, which was further expanded by PARP14 deficiency (Fig. 7f and Supplementary Fig. 13c).

To examine the relative contribution of PARP14 in the haematopoietic lineage to the lesion development after acute mechanical injury, we performed bone marrow transplantation from *PARP14*^+/+^ and *PARP14*^−/−^ mice. In lethally irradiated *PARP14*^+/+^ mice whose bone marrow was reconstituted by *PARP14*^−/−^ bone marrow (BMT^*PARP14*−/−→+/+^ mice), neointima formation after injury was accelerated, as compared with control *PARP14*+/+ mice whose bone marrow was reconstituted by *PARP14*^+/+^ cells (BMT^*PARP14*+/+→+/+^ mice; Fig. 7g). These results indicate that PARP14 derived from the haematopoietic cell lineage, the majority of which are macrophages in the neointima, plays an important role in the development of arterial disease.

### Haematopoietic PARP14 deficiency enhances atherogenesis

**Figure 8:**
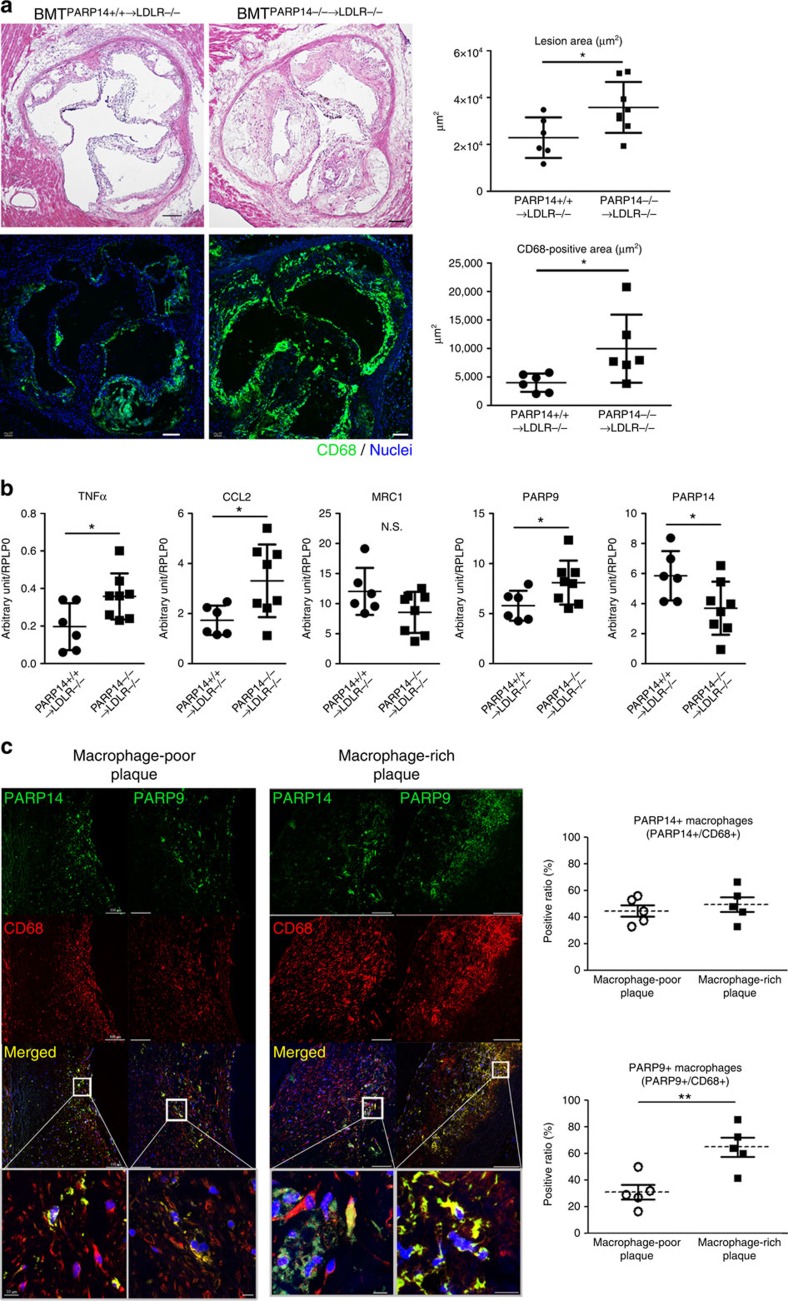
Haematopoietic PARP14 in mouse atheromata and PARP9–PARP14 expression in human plaques. (**a**) Representative image and quantification of aortic root lesion formation and CD68+ macrophage accumulation (green, Alexa 488) in high-fat and high-cholesterol diet-fed *LDLR*^−/−^ mice whose bone marrow was reconstituted by *PARP14*^−/−^ mice (BMT*^PARP14^*^−/−→*LDLR*−/−^ mice, *n*=5), compared with *LDLR*^−/−^ mice with *PARP14*^+/+^ bone marrow (BMT*^PARP14^*^+/+→*LDLR*−/−^ mice, *n*=6–7). Scale bars, 100 μm. (**b**) mRNA expression of the aorta from **a**. *n*=6–8. (**c**) Immunofluorescence staining of PARP14 and PARP9 proteins (green, Alexa 488) in human carotid plaques. CD68 (red, Alexa 594). Nuclei (blue, 4,6-diamidino-2-phenylindole, DAPI). Scale bars, 100 μm; insets, 10 μm (*n*=5). Prevalence of PARP14+ or PARP9+ macrophages in macrophage-poor versus macrophage-rich plaques. *P<0.05 and **P<0.01, respectively, by Student's *t*-test. Error bars indicate s.d.

To further examine the role of PARP14 in chronic arterial diseases, we used high-fat/high-cholesterol-fed low-density lipoprotein receptor-deficient (*LDLR*^−/−^) mice, an established model of atherosclerosis. Lethally irradiated *LDLR*^−/−^ mice whose bone marrow was reconstituted with *PARP14*^−/−^ cells (BMT^*PARP14*−/−→*LDLR*−/−^) or *PARP14*+/+ cells (BMT^*PARP14*+/+→*LDLR*−/−^) underwent high-fat/high-cholesterol feeding to develop chronic atherosclerotic plaques. Sixteen weeks after the initiation of an atherogenic diet, BMT^*PARP14*−/−→*LDLR*−/−^ mice exhibited more plaque formation and macrophage accumulation in the aortic root compared with BMT^*PARP14*+/+→*LDLR*−/−^ mice (Fig. 8a). The aorta of BMT^*PARP14*−/−→*LDLR*−/−^ contained higher expression levels of the pro-inflammatory TNFα and CCL2/MCP-1, while MRC1 expression tended to be lower (Fig. 8b). BMT from *PARP14*^−/−^ mice also increased PARP9 expression in the aorta (Fig. 8b). These results are consistent with other *in vitro* and *in vivo* data that we have reported in the present study. Taken together, these findings indicate that PARP14 derived from the haematopoietic lineage, the majority of which are macrophages in arterial lesions, plays a protective role against the development of arterial diseases, which verifies our prediction by network analysis.

### Macrophage-rich human atheroma and PARP9

Seeking additional *in vivo* evidence for the potential role of PARP9 and PARP14 in arterial disease involved immunohistochemical analysis of human carotid atherosclerotic plaques surgically removed by endarterectomy. PARP9 and PARP14 signals were predominantly localized in plaque macrophages, as indicated by the overlapping CD68-positive signal (Fig. 8c and Supplementary Fig. 14). Quantitative analysis demonstrated that more macrophages were immunoreactive for PARP9 in macrophage-rich plaques than in macrophage-poor plaques, while there was no significant difference in PARP14-positive macrophages (Fig. 8c). While some macrophages in the human plaques coexpressed PARP9 and PARP14, other cells stained positively for either PARP9 or PARP14 alone (Supplementary Fig. 14). These lines of *in vivo* evidence indicate that macrophages in arterial lesions are heterogeneous, which may reflect diverse levels of pro-inflammatory activation in individual cells.

### Single-cell analysis of primary human macrophages

The diversity in expression patterns of PARP9 and PARP14 in human plaques necessitated the additional assessment of macrophage subpopulations using single-cell gene expression profiling^38^ of PBMC-derived human CD14-positive macrophages. Examined cell numbers were 86 in M(-) and 84 in M(IFNγ) of Donor 1, 93 in M(-) and 86 in M(IFNγ) of Donor 2 and 90 in M(-) and 81 in M(IFNγ) of Donor3, respectively. Our examination investigated whether responses of human primary macrophages towards IFNγ stimulation are heterogeneous. Comparing average levels of readouts (for example, inflammation-related factors) in the entire group of cells by qPCR cannot address this question. A workflow of single-cell gene profiling is shown in Supplementary Fig. 15a.

**Figure 9:**
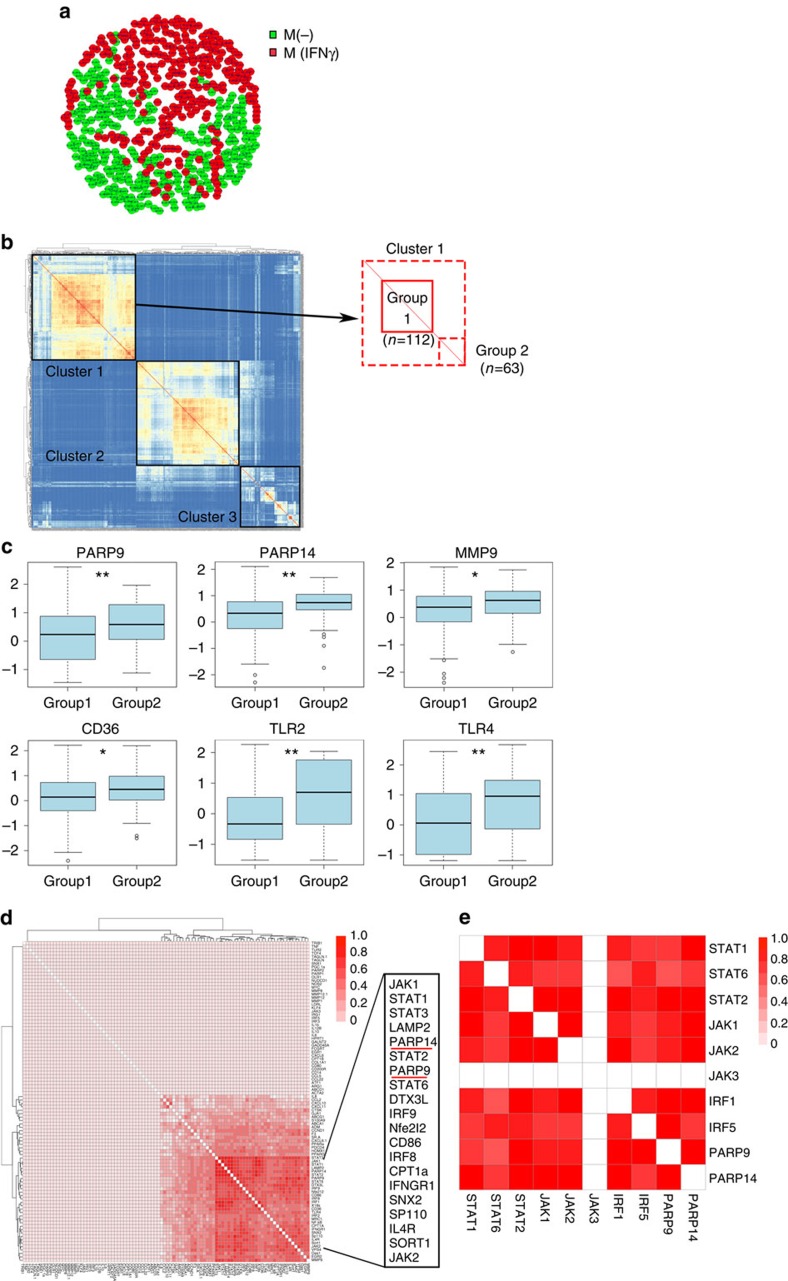
Single-cell gene expression analysis of CD14+ macrophages. (**a**) Heterogeneity in IFNγ-stimulated compared with unstimulated cells in combined all donor cells (*n*=520). (**b**) Similarity map of cells from all donors/conditions reveals three subpopulations; IFNγ-stimulated cells (Cluster 1), unstimulated cells (Cluster 2) and mixed populations (Cluster 3). Cluster 1 inset—there are two further subpopulations within IFNγ-stimulated cells, such as Groups 1 and 2. (**c**) The relative expression data for genes related to macrophage function are compared between Groups 1 and 2 (*n*=112 and *n*=63, respectively). (**d**) Similarity maps of 91 genes examined. (**e**) Gene similarity map of PARP9/14, STAT, JAK and IRF genes based on analysis with all cells from all donors and conditions. *P<0.05 and **P<0.01, respectively, by Student's *t*-test. Error bars indicate s.d.

The expression patterns of 91 target genes (Supplementary Table 4) in M(-) (*n*=268) and M(IFNγ) (*n*=252; *n*=520 in total) derived from the three different donors. All cells were evaluated against each other based on their gene expression dissimilarity (Manhattan distance, http://www.nist.gov/dads/). We present the distance matrix as a distance-based graph. By combining the two conditions—M(-) and M(IFNγ)—across three donors (six conditions in total), M(-) cells (green) and M(IFNγ) (red) are clearly segregated, forming distinct clusters (Fig. 9a). However, there are ‘trails' of M(IFNγ) that appear in the M(-) cluster, suggesting potential heterogeneity. Although this may be attributable to donor-to-donor variations, similar patterns were observed within individual donors as well (Supplementary Fig. 15b). As we are particularly interested in the expression profiles of PARP9 and PARP14, we examined variations/correlations in their expression levels in M(-) and M(IFNγ) human primary macrophages. In both phenotypes, PARP9 and PARP14 were correlated. However, M(-) cells showed lower levels of variation for PARP9 and PARP14 mRNA expression than did M(IFNγ) (Supplementary Fig. 15c). These findings suggest that the ‘IFNγ-polarized' human primary macrophages are heterogeneous.

It is possible to subdivide the cells into subpopulations based on expression profile similarity (Fig. 9b). Using Ward's linkage, we observed the following three subpopulations: M(IFNγ) (Cluster 1), M(-) (Cluster 2) and Mixed (Cluster 3). It is important to note that these populations are not homogeneous and can be further divided into at least two subgroups (Groups 1 and 2, Fig. 9b) in human primary M(IFNγ) (Cluster 1). Both PARP9 and PARP14 were significantly higher in Group 2 than in Group1. Considering the possible importance of PARP9 and PARP14 in the macrophage phenotype, we subsequently examined the genes related to macrophage functions in these two Groups 1 and 2. The potent matrix-degrading enzyme MMP-9 and the pattern recognition receptors CD36, TLR2 and TLR4 were higher in Group2, indicating that this subpopulation, which is associated with increased PARP9 and PARP14 expression, may possess a more pro-inflammatory phenotype (Fig. 9c). In our *in vitro* and *in vivo* experiments, RNA silencing or genetic deletion of PARP14 enhanced pro-inflammatory responses in IFNγ-stimulated macrophages. In the single-cell analysis, average levels of MMP-9 and TLR4 mRNA expression were also higher in IFNγ-stimulated macrophages treated with PARP14 siRNA (Supplementary Fig. 15d). However, their responses to PARP14 were heterogeneous.

The gene expression correlation matrix of genes across all cells (Fig. 9d) demonstrates that both PARP9 and PARP14 are closely associated with genes known to participate in IFNγ signalling (for example, JAK1, JAK2, STAT1, STAT6, IRF1 and IRF5). This correlation is highly specific to these genes, and does not extend to other genes that we tested in this assay. Genes that are not correlated with PARP9 and PARP14, among all 91 genes tested, include JAK3, a mediator of IL-4 signalling (Fig. 9e). Of interest, these correlations are further enhanced in M(IFNγ) compared with M(-) (Supplementary Fig. 15e).

## Discussion

The present study provides new evidence that PARP9 and PARP14 regulate macrophage activation. The specific novel findings demonstrated in this report include the following: (1) PARP9 promotes IFNγ-induced responses in mouse and human macrophages; (2) PARP14 suppresses IFNγ responses in mouse and human macrophages; (3) PARP14 induces IL-4-triggered responses in mouse and human macrophages; (4) PARP9 and PARP14 appear to have physical and functional interactions; (5) PARP9 and PARP14 closely interact with components of IFNγ signalling in macrophages; (6) PARP14-induced mono-ADP-ribosylation of STAT1 inhibits its phosphorylation; (7) the PARP9 and PARP14 interactomes have significant proximity to the coronary artery disease module; (8) PARP14 deficiency indeed accelerates macrophage activation and lesion development in mouse models of acute and chronic arterial diseases; (9) PARP14 in the haematopoietic lineage exerts protective effects against arterial diseases; and (10) single-cell analysis revealed that IFNγ-stimulated human primary macrophages derived from CD14+ PBMC contain subpopulations, in which PARP9 and PARP14 are closely associated with genes of IFNγ signalling. Our findings provide insight into new mechanisms for macrophage activation that play a critical role in the pathogenesis of inflammatory arterial diseases, a global health burden.

We aimed to establish the unambiguous evidence for the role of PARP9 and PARP14 in macrophage activation using *in vitro* and *in vivo* studies. In this study, M(IFN) and M(IL-4) as two multidimensional models of macrophage heterogeneity were used in an unbiased proteomics approach. We then validated our key results on the functionality of PARP9 and PARP14 in mouse and human primary macrophages. Bioinformatic analysis of single-cell gene profiling in human primary macrophages revealed close links among PARP9, PARP14 and IFNγ pathway-related molecules (for example, JAK1, JAK2, STAT1 and STAT6), suggesting that these PARP family members contribute critically to the process of M(IFNγ) activation.

We took advantage of the availability of a well-characterized paradigm of macrophage heterogeneity. The balance of diverse macrophage phenotypes (for example, pro-inflammatory versus anti-inflammatory subsets) may regulate normal homeostasis and disease mechanisms. Effective and safe anti-inflammatory therapies may require the fine-tuning of the imbalance of diverse macrophage phenotypes (for example, suppressing excessively activated pro-inflammatory macrophages without compromising protective functions or with enhancing anti-inflammatory activation). Our major goal was to identify molecules or pathways that may regulate the delicate balance of macrophage heterogeneity using global proteomics of M(IFNγ) and M(IL-4). We thus used stringent criteria to choose proteins that increased during M(IFNγ) activation and decreased in M(IL-4). Our *in vitro* experiments indicate that the interplay between PARP9 and PARP14 may indeed regulate the M(IFNγ)/M(IL-4) balance. We further validated the *in vitro* results in human arterial lesions and mouse models of arterial disorders, neither of which is solely regulated by IFNγ or IL-4, to provide clinically translatable evidence. Human and mouse atherosclerotic plaque macrophages express PARP9 and PARP14. We identified four macrophage subpopulations in human lesions: PARP9+/PARP14+; PARP9+/PARP14−; PARP9-/PARP14+; and PARP9−/PARP14−. Single-cell gene expression analysis further revealed that IFNγ-stimulated human primary macrophages—M(IFNγ)—are heterogeneous, which may support an emerging concept of the multidimensional model of macrophage activation^21^. In addition, subpopulations within these IFNγ-stimulated cells may have different functions, as shown by associations with MMP-9, CD36, TLR2 and TLR4. Future studies addressing the functional significance of the heterogeneity in human primary macrophages may lead to the development of personalized medical solutions.

To explore the evidence for the anti-atherogenic role of PARP14 beyond *in vitro* assays, we used *PARP14*^−/−^ mice. Genetic deletion of PARP14 indeed promoted macrophage activation and accumulation in the intima of mechanically injured arteries, offering *in vivo* proof of concept. Moreover, significant acceleration of acute arterial lesion formation and chronic atherosclerosis was observed in the mice reconstituted with *PARP14*^−/−^ bone marrow. These findings suggest that haematopoietic PARP14 plays a central role in arterial disease.

Our study further revealed the new biology for understudied members of the PARP family—PARP9 and PARP14. ADP-ribosylation assays demonstrated that PARP14 has the ability to mono-ADP-ribosylate STAT1 and mass spectrometric analysis identified two ADP-ribosylation sites proximal to its key phosphorylation site. Interestingly, PARP9 suppresses PARP14-dependent mono-ADP-ribosylation of STAT1. Furthermore, mutations of STAT1α ribosylation sites for PARP14 enhanced its phosphorylation and pro-inflammatory gene expression in macrophages. The interplay of PARP9 and PARP14 in STAT1α ADP-ribosylation and phosphorylation may in part explain why PARP9 and PARP14 exert opposing effects on IFNγ- and IL-4-induced macrophage activation. Supplementary Fig. 16 demonstrates an overview of regulatory mechanisms for the IFNγ-STAT1 and IL-4-STAT6 pathways by PARP9 and PARP14.

In this study, unbiased global proteomics and bioinformatics of IFNγ- and IL-4-treated macrophages implicated PARP9 and PARP14 in novel mechanisms of macrophage activation. The subsequent network-based prediction of the close relationship between the macrophage PARP14–PARP9 module and human coronary artery disease genes supported the premise that our proteomics screen would effectively identify regulators of macrophage activation in the context of cardiovascular diseases. The approach was then followed by a series of *in vitro* and *in vivo* analyses on mouse and human cells/tissues that demonstrated the novel concept that PARP9 promotes and PARP14 suppresses IFNγ-induced activation of macrophages (Supplementary Fig. 16). The present study also represents our strategy of target discovery research assisted by global proteomics screening and subsequent validation studies (Supplementary Fig. 17). Collectively, our discoveries indicate that inhibition of PARP9 and/or activation of PARP14 may attenuate macrophage-mediated vascular diseases, and also provide new insight into the development of effective therapies for other inflammatory disorders.

## Methods

### Cell stimuli for cell culture and TMT sample preparation

In this study, we used mouse and human IFNγ 10 ng ml^−l^ and IL-4 10 ng ml^−1^ (R&D systems) as stimuli for macrophage activation, respectively. The murine monocyte/macrophage cell line RAW264.7 was obtained from American Type Culture Collection (ATCC, TIB-71, Rockville, MD) and maintained in 10% fetal bovine serum (FBS, Life Technologies) containing DMEM (Sigma) supplemented with penicillin and streptomycin (Corning) at 37 °C in humidified 5% CO_2_. Before stimuli (IFNγ 10 ng ml^−1^ and IL-4 10 ng ml^−1^), cells were starved for 24 h with 0.1% FBS-containing media. THP-1 was also purchased from ATCC (TIB-202) and maintained in RPMI 1640 medium in 10% FBS with penicillin and streptomycin at 37 °C in humidified 5% CO_2_. The macrophage-like state was obtained by treating the THP-1 monocytes for 48 h with PMA (200 ng ml^−1^, Sigma). Mycoplasma contamination test was routinely performed (once a month).

Each cell culture experiment, unstimulated, INFγ and IL-4, was prepared for isobaric labelling using the 6-plex TMT strategy (Pierce). For sample preparation the cells were lysed and proteolysed (Lys-C, Wako Chemicals) using the in-solution urea+ RapiGest (Waters) strategy detailed previously (Supplementary Fig. 1a)^39^. Tryptic peptides were labelled with TMT 6-plex reagent (Pierce), combined and desalted using Oasis Hlb 1cc (10 mg) columns (Waters). The peptides were then fractionated into 24 fractions based on their isoelectric focusing point (pH range of 3–10) using the OFF-gel system (Agilent; Supplementary Fig. 1b). The fractions were dried using a tabletop speed vacuum (Fisher Scientific), cleaned with the Oasis columns and resuspended in 40 μl of 5% acetonitrile (Fisher Scientific) and 5% formic acid (Sigma-Aldrich) for subsequent analysis by liquid chromatography/mass spectrometry (LC/MS).

### Liquid chromatography tandem mass spectrometry

The high-resolution/accuracy LTQ-Orbitrap Elite (Thermo Scientific) analysed TMT peptide samples, and the Q Exactive (Thermo Scientific) and Elite analysed *in vitro* ribosylated peptides. Both mass spectrometers are fronted with a Nanospray FLEX ion source, and coupled to an Easy-nLC1000 HPLC pump (Thermo Scientific). The peptides were subjected to a dual column set-up: an Acclaim PepMap RSLC C18 trap column, 75 μm × 20 cm (50 μm × 15 cm on the Q Exactive), and an Acclaim PepMap RSLC C18 analytical column 75 μm × 250 mm (Thermo Scientific). For TMT analysis the analytical gradient was run at 250 nl min^−1^ from 10 to 30% Solvent B (acetonitrile/0.1% formic acid) for 90 min, followed by 5 min of 95% Solvent B. Solvent A was 0.1% formic acid. For ribosylated peptides the gradient was run at 250 nl min^−1^ from 5 to 28% Solvent B for 10 or 30 min, followed by 5 min of 95% Solvent B. All reagents were HPLC-grade. For TMT analysis, LTQ-Orbitrap was set to 120 K resolution and the top 20 precursor ions (within a scan range of 380–2,000 *m*/*z*) were subjected to higher-energy collision-induced dissociation (HCD, collision energy 40%, isolation width 3 *m*/*z*, dynamic exclusion-enabled, starting *m*/*z* fixed at 120 *m*/*z* and resolution set to 30 K) for peptide sequencing (MS/MS). The Q Exactive was set to 140 K resolution with a top 10 precursor selection method (scan range of 380–1,500 *m*/*z*). HCD was set to a stepped normalized collision energy of 25±10%, isolation width of 1.6 *m*/*z*, dynamic exclusion-enabled and resolution set to 17.5 K for MS/MS. Ribosylated peptide candidates were screened in the MS/MS scan by the m6 peak of 348.1 (refs 40, 41). Unmodified forms were calculated by subtracting the mass of the ADP-ribose (541.06 Da) from the observed precursor. Modified and unmodified *m/z* values and corresponding retention time windows were submitted to an inclusion list and analysed in using the data-independent acquisition module of the Q Exactive (*R*=35 K).

The MS/MS data were queried against the mouse or human UniProt database (downloaded on 27 March 2012) using the SEQUEST search algorithm via the Proteome Discoverer (PD) Package (version 1.3, Thermo Scientific)^42^, using a 10 p.p.m. tolerance window in the MS1 search space and a 0.02 Da fragment tolerance window for HCD. Methionine oxidation was set as a variable modification, and carbamidomethylation of cysteine residues and 6-plex TMT tags (Thermo Scientific) were set as fixed modifications. The peptide false discovery rate (FDR) was calculated using Percolator provided by PD: the FDR was determined based on the number of MS/MS spectral hits when searched against the reverse, decoy mouse or human database^43^,^44^. Peptides were filtered based on a 1% FDR. Peptides assigned to a given protein group, and not present in any other protein group, were considered as unique. Consequently, each protein group is represented by a single master protein (PD Grouping feature). Master proteins with two or more unique peptides were used for TMT reporter ratio quantification (Supplementary Table 2 contains a summary of peptides and peptide-spectrum matches (PSMs) for PARP9 and PARP14). Ribosylation spectra were manually annotated^40^,^41^.

### Proteomics normalization and filtering strategy

For each PSM the TMT ion channel intensities were normalized to the time-zero channel (reference normalization, for example, 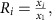 where *i*=1, 2, …, 6 and *x*_1_ is the time-zero abundance). The protein's abundance was then calculated by taking the median of its corresponding PSM ratios^45^. To extract the proteins that increase in IFNγ-stimulated (M(IFNγ)) but decrease in IL-4-stimulated condition (M(IL-4)), we applied a simple filtering logic that exploited the available unstimulated control data set. In this study, unstimulated control, M(-), serves as a control for the biological signal owing to the cell culture condition; that is, protein traces M(IFNγ) and M(IL-4) that exceed the edges of the baseline are more likely to be bona fide IFNγ- and IL-4-specific responses (Supplementary Fig. 3b). Protein profiles whose abundances surpassed the baseline, which was defined as maximum relative abundance at 8 h of M(-) (+0.13, log10 of relative abundance), after supplement of INFγ established the general threshold for the entire time M(IFNγ) data set. This cutoff value was the same that was used for both RAW264.7 and THP-1 M(IFNγ) data sets. Moreover, the proteins extracted from the IFNγ-stimulated filtering step were cross-referenced not only to M(-) but also to M(IL-4) data sets where proteins were expected to possess opposite profiles with respect to M(IFNγ) (Supplementary Fig. 3b). Therefore, the final list of candidate proteins (Fig. 1b) had profiles whose abundances increased in M(IFNγ) and decreased in M(IL-4), respectively, beyond their baseline controls.

### Proteomics data clustering

Clustering was performed using the model-based algorithm^46^ in R, which is based on finite mixture models; as such, it can successfully be applied to time series data analyses^44^ such as those acquired for M(IFNγ) and M(IL-4)^47^ (Supplementary Fig. 3c). In this approach, each time series (protein profile) y*i*, *i*=1, 2, …, *N* (where *N* is the number of TMT channels) is considered to be a single entity connected by a line. Clustering is achieved for a traditional finite mixture model by assigning each time series, y*i*, to a cluster. The non-supervised model-based clustering uses the Expectation Maximization algorithm to assign the profile to a specific cluster. MCLUST has the advantage of using the Bayesian Information Criteria to determine the number of clusters that best partition the data set by maximizing the intradata set variability. Finally, stronger covariance (and thus also dependencies) between sets of two time points is enabled by sum normalization^48^. After clustering was performed (Supplementary Fig. 4a) we focused on proteins shared in all three data sets (unstimulated control, IFNγ-stimulated and IL-4-stimulated) for both RAW264.7 and THP-1 cells, with the purpose of identifying proteins whose profiles increased in IFNγ-stimulated condition and decreased in IL-4-stimulated, but maintained at basal levels in unstimulated control condition. We inspected all clusters looking for an increase in the relative abundance of proteins with respect to time zero at any time point in IFNγ-stimulated, and subsequently cross-referenced the IL-4-stimulated and unstimulated control clusters for proteins whose profiles decreased and remained within the baseline, respectively. The proteins that fulfilled these three criteria are shown in the heat maps for RAW264.7 and THP-1 cell data sets (Supplementary Fig. 4b). Finally, to further narrow the list of proteins of interest, we prioritized those that were common to both species (Fig. 1c), as determined by identical Uniprot protein IDs. Heat maps were used only with the aim of ordering the protein expression levels according to the row obtained *Z*-score (that is, *z*=(*x*-mean)/s.d.). The proteins were clustered in the horizontal direction hierarchically using Euclidean distance and average linkage methods (Fig. 1c and Supplementary Fig. 4b). In these data matrices, each column represents the TMT time point analysis 0, 8, 12, 24, 48 and 72 h, and each row corresponds to a protein gene ID.

### PARP14–PARP9 network analysis

As a parallel approach to further investigate the candidacy of PARP9 and PARP14 as cardiovascular and metabolic diseases, we turned to *in silico* or network-based prediction methods under the premise that a potential PARP14–PARP9 interactome would be close in vicinity to its pertinent vascular disease network/module.

To evaluate the impact of PARP14–PARP9 neighbours, we used the HumanNet interaction database^49^. We then used the random-walk methodology^50^ to construct the disease modules from the functional database using gene–disease associations extracted from genome-wide association studies and from the OMIM ( http://www.ncbi.nlm.nih.gov/omim) and the MalaCards ( http://www.malacards.org) databases for: Cardiomyopathy, Coronary Heart disease, Heart Failure, Hypercholestermia, Hypertension, Metabolic Traits, Osteoporosis, Cardiovascular Risk Factors, Sudden Cardiac Arrest, Systemic lupus erythematosus, Sjögren's syndrome, polymyositis and dermatomyositis. The PARP9–PARP14 module was determined by first neighbours in the functional database. Further, we restricted the first neighbours to those that are expressed in macrophages based on gene expression. The motivation of this network analysis is based on two recent studies of ours that investigated the localization of disease in the network^51^ and measure the separation of disease modules in the network^52^, both of which have proved instrumental in identifying disease modules. These network topology-based methods have been rigorously validated in both of these peer-reviewed publications by means of biological enrichment as well as compared with other network-based methodologies. On the basis of this selection, we considered 55 first neighbours for PARP9 and 149 for PARP14 that we annotated collectively as the PARP9–PARP14 module. Further, we measured the closeness of the first neighbours using the shortest-path topology measure with the disease modules above. For each disease module, we calculated the shortest-path distances to the PARP9–PARP14 module and compared these distances to the random distance distribution with the same module size (Wilcoxon test and Benjamini–Hochberg correction for multiple testing). Finally, we verified that the module size does not affect the significance of the *P* values for the Coronary Heart Disease and Osteoporosis modules by recalculating the random distances by reducing the module size to the top 35 genes from random walk.

### Mouse peritoneal macrophages

Four days after intraperitoneal injection of 2.5–3 ml of 4% thioglycollate (Fisher Scientific), peritoneal macrophages were obtained by injecting 5 ml of ice-cold PBS into the peritoneal cavity using 25 G needle, followed by collection of the fluid using 23 G needle. In all, 5 × 10^5^ cells were cultured on 24-well plates (Corning) with RPMI containing 10% FBS. Nonadherent cells were discarded after 16 h. After washing with PBS, cells were incubated with IFNγ 10 ng ml^−1^ and IL-4 10 ng ml^−1^ for 24 h until harvesting.

### Primary mouse macrophages derived from BMCs

BMDMs were isolated and differentiated as described previously^53^. Briefly, whole-BMCs were harvested from femurs of 10–12-week-old male *PARP14*^−/−^ or *PARP14*^*+/+*^ mice (C57BL/6 mice, Jackson Laboratory) under aseptic conditions and cultured in RPMI 1640 (Lonza) supplemented with 10% FBS, penicillin and streptomycin (Corning) in presence of macrophage colony-stimulating factor (M-CSF; 50 ng ml^−1^; Peprotech) at 37 °C in humidified 5% CO_2_. After 7-day culture, cells were incubated with IFNγ 10 ng ml^−1^ and IL-4 10 ng ml^−1^ for 24 h until harvesting. After 7 days of culture, cells were incubated with IFNγ 10 ng ml^−1^ and IL-4 10 ng ml^−1^ for 24 h. Whole-BMCs were cultured in M-CSF (50 ng ml^−1^) for 7 days and analysed. FACS analysis showed that most of cells (>80%) were positive for CD11b, CD115 and weakly positive for F4/80 and negative for CD11c and Ly6G, indicating the differentiation of BMCs into macrophages. FACS was performed on the FACSAria2 (BD Bioscience), and data were analysed using the Flowjo software (Tree Star).

### Isolation of CD14+ human PBMCs

PBMCs were isolated from buffy coat using lymphocyte separation medium (MP Biomedicals). After isolation of PBMCs, CD14^+^ cells were isolated by Dynabeads in combination with anti-CD14 antibody (Dynabeads FlowComp human CD14, Invitrogen), according to the manufacturer's instruction. Briefly, CD14+ dynabead-bound cells (CD14^+^ cells) were isolated using a magnet. After releasing Dynabeads from cells, bead-free CD14^+^ PBMCs (5 × 10^5^ cells) were cultured in 24-well dish plates with 0.5 ml of RPMI supplemented with 10% FBS at 37 °C in 5% CO_2_.

### Immunohistochemistry and immunofluorescence

Samples were cut into 7 μm thin slices, and cryo-sections were fixed in acetone. After blocking in 4% appropriate serum, sections were incubated with primary antibodies (Mac3 (1:200, M3/84, BD Pharmingen), PARP14 (1:50, HPA01206, Sigma-Aldrich), PARP9 (1:100, ab53796, Abcam) and human CD68 (1:200, M0876, Dako), followed by biotin-labelled secondary antibody (1:250, Vector Laboratories, Burlingame, CA, USA) and streptavidin-coupled Alexa Fluor 488 antibody (Life Technologies). For immunofluorescence double labelling, after avidin/biotin blocking (Vector Laboratories), the second primary antibody was applied overnight at 4 °C, followed by biotin-labelled secondary antibody and streptavidin-coupled Alexa Fluor 594 antibody (1:250, Life Technologies). Sections were washed in PBS and embedded in mounting medium containing 4,6-diamidino-2-phenylindole (Vector Laboratories). For bright-field immunohistochemistry on tissue sections, following the first biotin-labelled secondary antibody incubation, sections were incubated with streptavidin-labelled horseradish peroxidase (HRP) solution (Dako), followed by 3-amino-9-ethylcarbazole (AEC) solution. Slides were examined using the Eclipse 80i microscope (Nikon, Melville, NY, USA) or the confocal microscope A1 (Nikon). All images were processed with the Elements 3.20 software (Nikon).

### Human tissue and counting PARP14–PARP9-positive macrophages

Atherosclerotic carotid arteries (*n*=10) were collected from patients undergoing endarterectomy procedures at Brigham and Women's Hospital according to IRB protocol # 1999P001348 (PL). Informed consent was not necessary because all samples are considered as ‘discarded material.' Samples have two groups, such as macrophage-rich (*n*=5) and no macrophage-rich (*n*=5) plaques, respectively. Samples were embedded in optimal cutting temperature (OCT) compound and stored at −80 °C until use. In three different fields of each sample (*n*=5), 200 cells (nuclei) with CD68-positive cells were evaluated whether they express PARP14 and/or PARP9 (600 cells per sample). The quantification of immunofluorescence was performed by examiners who were blinded to group allocation (macrophage-rich versus no plaque).

### Animal ethics

All animal experiments used in this study were approved by and performed in compliance with Beth Israel Deaconess Medical Center's Institutional Animal Care and Use Committee (Protocol 024-2014).

### PARP14-deficient mice

*PARP14*^−/−^ mice were backcrossed into the C57BL/6 genetic background over 10 generations^25^,^54^. Male *PARP14*^−/−^ mice and age-matched *PARP14*^+/+^ mice were used for a vascular injury model or as donors for BMT.

### Wire-induced acute vascular injury

Transluminal arterial injury was induced for 10-week-old mice by inserting spring wire (0.38 mm in diameter, C-SF-15-15, Cook) into the femoral artery under microscopic observation (leica M80)^55^. Right common femoral artery, superficial femoral artery and deep femoral artery (DFA) were first exposed. After looping SFA with 9-0 nylon suture, a small hole in the DFA was made and a wire was inserted through the hole and advanced to the iliac artery (10 mm). DFA was ligated with 9-0 nylon suture when the wire was removed at the closer point to DFA and common femoral artery bifurcation than the hole.

### Bone marrow reconstitution

Bone marrow was reconstituted as described previously^56^ with minor modifications. Briefly, recipient mice were lethally irradiated with a total dose of 1,000 rads. The next day, unfractionated bone marrow cells (BMCs, 1 × 10^6^) that had been harvested from donor mice and suspended in 0.3 ml of phosphate-buffered saline were administered to each recipient mouse via the tail vein. We confirmed bone marrow reconstitution by examining gene expression of PARP14 in the bone marrow of BMT^*PARP14*+/+→+/+^ and BMT^*PARP14*−/−→+/+^ mice (93.3% reduction of the *PARP14* gene in BMT^*PARP14*−/−→+/+^ compared with BMT^*PARP14*+/+→+/+^ mice). Four weeks after bone marrow reconstitution, wire-mediated femoral artery injury was performed. Two weeks after injury, to collect arterial samples, mice were killed by intraperitoneal administration of an overdose of Pentobarbital and then perfused with 0.9% NaCl solution at a constant pressure via the left ventricle^57^.

### High-fat diet-induced chronic atherosclerosis

First, we examined PARP9 and PARP14 expression in Apolipoprotein E-deficient (*ApoE*−/−) mice, which were backcrossed with C57BL6 mice over 10 generations and then fed by high-fat and high-cholesterol diet for 20 weeks. As a chronic atherosclerosis model, we used low-density lipoprotein receptor (LDLR)-deficient mice (*LDLR*−/−) fed a high-fat and high-cholesterol diet for 16 weeks. Bone marrow of *PARP14*−/− or sex and age-matched *PARP14*+/+ mice was transplanted to lethally irradiated LDLR−/− mice, as described above (BMT^*PARP14*+/+→*LDLR*−/−^ and BMT^*PARP14*−/−→*LDLR*−/−^ mice). These mice were fed a high-fat and high-cholesterol diet for 16 weeks after bone marrow reconstitution, and tissues were harvested for histological and molecular analyses.

### Evaluation of arterial lesions

The collected tissue was embedded in OCT compound (Tissue-Tek) and then frozen in liquid nitrogen and stored at −80 °C until further use. Intima and media area and their ratio in injured femoral arteries were measured by manual tracing in at least three sections of three different levels with 100 μm interval in each animal (*n*=5), using the Elements 3.20 software (Nikon). The quantification of histology and immunohistochemistry was performed by examiners who were blinded to group allocation (*PARP14*+/+ versus *PARP*14−/− and BMT^*PARP14*+/+→+/+^ versus BMT^*PARP14*−/−→+/+^ and BMT^*PARP14*+/+→*LDLR*−/−^ versus BMT^*PARP14*−/−→*LDLR*−/−^ mice). No randomization method was used.

### RNA interference

RNA silencing was performed as described previously^58^. Briefly, 20 nmol l^−1^ siRNA against PARP14 (L-023583 for human cells and L-160447 for mouse cells) and PARP9 (L-014734 for human cells and L-05024901 for mouse cells; all ONTARGETplus SMART-pool, Thermo Scientific) or non-targeting siRNA (scramble control siRNA, ON-TARGET Non-Targeting Pool, Thermo Scientific) was transferred into macrophages using SilenceMag (BOCA Scientific, Boca Raton, FL), according to the manufacturer's instruction. Target sequences of siRNA pool were follows:

Human PARP14: 5′-UAAUCAAAGGUCUCUUAUG, UAAUGCUUAAGGUCCUCAU, UCAUUAUACUGCCAUUCUA-3′ and 5′-GAACUCUUGACAUCAUUUC-3′,

Human PARP9: 5′-AAUUACAUCUGCCGUCUGC-3′, 5′-UUUGUGGCAAGAAAUUCCG-3′, 5′-UUAAUCAACAGGGCUGCCA-3′ and 5′-UACAGCCAAACUUAUUCUG-3′

Mouse PARP14: 5′-CUUGAAAGCUUUACGUAUA-3′, 5′-CAGCAAUAGGAACGGGAAA-3′, 5′-CCAAAGAACUUGAUCAACA-3′ and 5′-CGUAGUAGCAAAAGCGAUA-3′,

Mouse PARP9: 5′-ACACAAUGUCUUCGAAAUU-3′, 5′-CCAGACAGCUAUCGAAUUA-3′, 5′-CCAAAUAUGAUCUACGCAU-3′ and 5′-CGUACACAUUUCAACGAUA-3′.

Control scramble: 5′-UGGUUUACAUGUCGACUAA-3′, 5′-UGGUUUACAUGUUGUGUGA-3′, 5′-UGGUUUACAUGUUUUCUGA-3′ and 5′-UGGUUUACAUGUUUUCCUA-3′.

### Cell growth and cell viability

Cell proliferation, viability and apoptosis were assessed using the CellTiter 96 AQueous Nonradioactive Cell Proliferation Assay Kit (MTS), Cell Titer Blue assay and Apo-ONE Homogeneous Caspase-3/7 Assay Kit, respectively (Promega), according to the manufacturer's instructions.

### RNA preparation and real-time PCR

Total RNA from the cell culture was isolated using TriZol (Life Technologies), and reverse transcription was performed using the QuantiTect Reverse Transcription Kit (Qiagen, Hilden, Germany). The mRNA expression was determined by TaqMan-based real-time PCR reactions (Life Technologies). The following TaqMan probes were used: Hs99999902_m1 (human RPLP0), Mm00725448_s1 (mouse RPLP0), Hs00981511_m1 (human PARP14), Mm00520984_m1 (mouse PARP14), Hs00967084_m1 (human PARP9), Mm00518778_m1 (mouse PARP9), Hs00174128_m1 (human TNF), Mm00443258_m1 (mouse TNF), Hs00174097_m1 (human IL-1β), Mm01336189_m1 (mouse 1L1β), Mm00475988_m1 (mouse ARG1), Hs00267207_m1 (human MRC1), Mm00485148_m1 (mouse MRC1), Mm00440502_m1 (mouse NOS2) and Hs00234140_m1 (human CCL2). The expression levels were normalized to RPLP0. Results were calculated using the Delta-Delta Ct method, and presented as arbitrary unit.

### LCM and RNA amplification

LCM was performed on the Leica LMD6500 Microdissection System. Neointima were cut using the following LCM parameters: power, 50 mW; pulse duration, 2 ms; and spot size, 20 Hm. RNA was isolated using the PicoPure RNA Isoaltion Kit, followed by RNA amplification using the RiboAmp HS Plus RNA Amplification Kit (both Arcturus, Mountain View, CA, USA), according to the manufacturer's protocol. PCR array was performed using the Fluidigm PCR system.

### Western blot analysis

Cells were lysed with RIPA buffer containing protease inhibitor (Roche). Protein concentration was measured using the bicinchoninic acid method (Thermo Scientific). Total protein was separated by 8–10% SDS–PAGE and transferred using the iBlot western blotting system (Life Technologies). Primary antibodies against human and mouse PARP14 (1:250, HPA01206 Sigma-Aldrich), human and mouse PARP9 (1:250, ab53796, Abcam), human and mouse STAT1 (1:1,000, #9172, Cell Signaling), phosphorylated STAT1 (1:1,000, #9167, Cell Signaling), human and mouse STAT6 (1:2,000, #9362, Cell Signaling), mouse (1:1,000, ab54461, Abcam) and human (1:2,000, #9361, Cell Signaling) phosphorylated STAT6 and human and mouse β-actin (1:5,000; Novus) were used. For secondary antibodies, we used anti-mouse (1:1,000–5,000, A4416, Sigma) and rabbit (1:1,000–5,000, NA934-1ML, GE Healthcare Life Sciences) IgG antibodies. Protein expression was detected using Pierce ECL Western Blotting substrate reagent (Thermo Scientific) and ImageQuant LAS 4000 (GE Healthcare). Uncropped images of western blots are demonstrated in Supplementary Figs 18–20.

### ELISA

The amounts of TNFα and IL-1β released into the culture media after stimulation were measured using an ELISA kit following the manufacturer's instructions (Duoset Kit, R&D). The culture medium of unstimulated macrophage was used as the negative control. Standard, control or sample solution was added to the ELISA-well plate, which had been pre-coated with specific monoclonal capture antibody. After being shaken gently for 3 h at room temperature, the polyclonal anti-TNFα antibody, conjugated with horseradish peroxidase, was added to the solution and incubated for 1 h at room temperature. A substrate solution containing hydrogen peroxidase and chromogen was added and allowed to react for 20 min. The levels of cytokines were assessed by a plate reader at 450 nm and normalized with the abundance of standard solution.

### Nitrate quantification

To quantify nitric oxide in cell culture media of macrophages, mimicking iNOS concentration, the Griess Reagent Kit (G-7921, Life Technologies) was used.

### Co-IP

Cells were lysed in IP lysis buffer (Thermo Scientific). A volume of 100 μg of protein was incubated with PARP14 antibody (5 μg, Invitrogen) and Dynabeads streptavidin (Life Technologies) by rotation overnight at 4 °C, followed by washing three times with PBS/Tween 20 (0.02%), using a magnet to collect the beads after each wash. Five per cent of the precipitated protein sample was subjected to SDS–PAGE. Protein expression was detected using Pierce ECL western blotting substrate Reagent and ImageQuant LAS 4000.

### ADP-ribosylation assays

Recombinant human PARP14 and PARP9 (BPS Bioscience Inc.) proteins and BSA (Sigma-Aldrich) were incubated with recombinant human STAT1α (OriGene Technologies Inc.) or STAT6 protein (Sino Biological Inc.) at a final concentration of 5 ng μl^−1^ in the presence of 100 μM β-nicotinamide adenine dinucleotide hydrate (NAD; Sigma-Aldrich) or 6-biotin-17-NAD (Trevigen Inc.) in 50 mM Tris-HCl buffer (pH 7.4) for 1 h at room temperature. Ribosylation of STAT1α and STAT6 was detected by liquid chromatography tandem mass spectrometry (LC-MS/MS) after trypsin digestion (using the biotin-free NAD reaction) or by western blotting using Streptavidin-HRP (Abcam) after SDS–PAGE. Quantification of the relative abundances of ribosylated STAT1α peptides was completed by calculating the area under the curve (AUC) of the extracted ion chromatograms of the monoisotopic peaks of the modified versus unmodified peptides. The ratios were reported as AUC mod./(AUC mod. + AUC unmod).

### Construction and enforced expression of mutant STAT1

Human pcDNA-GFP-STAT1 was purchased from Addgene (Cambridge, MA, USA). Step-wise mutations (glutamic acid, E, to glutamine, Q) was introduced at the two ribosylation sites flanking the phosphorylation at Tyr701—E657 and E705—by recombinant PCR mutagenesis. Mutated constructs were verified by DNA sequencing. Mouse bone marrow macrophages were differentiated from bone marrow stromal cells using 10 ng ml^−1^ M-CSF for 12 days. pcDNA-GFP-STAT1 (WT) and the mutant pcDNA-GFP-STAT1 E657Q, E705Q, were transferred by Magnetofection (OzBioscience, San Diego, CA, USA). HEK293 cells were transfected using Lipofectamine LTX (Invitrogen, USA). Twenty-four hours after transfection, cells were serum-starved (0.1% FBS) for 2 h and stimulated with IFNγ for 1 h (phospho-STAT1) or 24 h (mRNA expression of pro-inflammatory factors). The overexpressed STAT1 was immunoprecipitated using anti-GFP antibody, clone 9F9.F9 (1:1,000, ab1218, Abcam). STAT1 phosphorylation at Tyr 701 was detected by anti-phospho-STAT1 (Tyr701; 1:1,000, mAb #7649, Cell Signaling). Antibodies against STAT1 (ab3987, Abcam) and GFP (ab290, Abcam) served as loading controls. Transfection into THP-1 cells was performed using the magnetofection method described above.

### Flow cytometry

The spleen was removed and homogenized to isolate splenocytes. Splenocytes were incubated in red-blood-cell lysis buffer (ACK lysing buffer, Gibco) to remove erythrocytes. After incubation with anti-mouse CD16/CD32 mAb (BioLegend, 0.5 μg per million cells) to block the Fc receptor, cells were then stained with antigen-presenting cell-conjugated CD11b, fluorescein isothiocyanate-conjugated Ly6c and phycoerythrin-conjugated Ly6G (BioLegend) in autoMACS running buffer containing bovine serum albumin, EDTA, PBS and 0.09% azide (Miltenyl Biotec) for 30 min. After washing cells with autoMACS running buffer, stained cells were analysed by FACSAria2 (BD Bioscience) and Flowjo software (Tree Star). Ly6c expression was evaluated in CD11b+Ly6G− splenic monocytes (apparent b).

### Single-cell gene expression analysis

For single-cell analysis of CD14+ PBMCs derived from three donors, cell capture and target pre-amplication steps were performed using the C1 system (Fluidigm) according to the manufacturer's instructions. Quantitative real-time PCR was performed using the BioMark 96.96 Dynamic Array platform (Fluidgm)^38^. After isolation of CD14+ PBMCs from buffy coat, cells were cultured for 10 days. Cells in unstimulated control condition were harvested on day10 and IFNγ-stimulated cells were harvested on day 11 after 24 h incubation with IFNγ 10 ng ml^−1^. The cell capture rate by the C1 chip was 89.6% (86/96) in unstimulated control and 83.3% (84/96) in IFNγ-stimulated of donor 1, 96.9% (93/96) in unstimulated control and 89.6% (86/96) in IFNγ-stimulated of donor 2, and 92.7% (89/96) in unstimulated control and 85.4% (82/96) in IFNγ-stimulated of donor3.

The raw real-time PCR reads for each array were transformed into *n* × m matrices using Python's Pandas libraries ( http://pandas.pydata.org/; where *n*=cell index and m=gene). Each data matrix was then processed and analysed using an in-house developed platform.

We first run a check for each column (genes) to see whether there are undetected values (CT=999) interspersed among positive reads (CT<25). If less than 10% of the reads are positive, we substitute those values with 999, and consider the entire gene undetectable for this array. On the other hand, if more than 10% of the genes are positive, their corresponding reads with undetected values are substituted with the average value of the positive reads. Granted, more sophisticated missing-value imputation (MVI) techniques exist^59^; however, we may not have enough features (96 features) to fully benefit from MVI nor is it clear whether this necessarily leads to improvement in signal. Moreover, genes requiring missing-value estimation tend to fall near the limit of detection, and are unlikely to benefit fully from MVI. For the purpose of having a fully populated matrix with no missing values, the averaged value therefore should suffice. The final missing-value adjusted reads are converted into log2exp via the following equation (equation 1), where LOD stands for the limit of detection and set at recommended default value of 24.


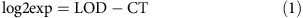


Although we do not normalize using housekeeping genes (see below for normalization method), they can be good indicators of the overall read quality for a given cell. Cells without the housekeeping gene expression (that is, *GAPDH*) were removed from analysis. Next, we calculate the mean expression value of the *GAPDH* gene (average log2exp of all cells for *GAPDH*). Cells with outlier *GAPDH* expression (more than 3 s.d.'s from the mean) were also excluded from the analysis.

Individual cells may exhibit extreme reads because of transcriptional burst, or because of high-instrument sensitivity [5]. Such extreme values need to be contained. Trimming methods (removal of extreme outliers based on the 5% quantile on either side of the read distribution) are unsuitable, as they would alter the dimensions of the matrix as well as lead to the loss of data. For example, given 100 genes with 100 observations each. If there is a 5% chance for every gene that at least one observation would contain an extreme outlier, then via the process of trimming, only 95 of the observations could be used for analysis since the outliers, and hence the entire column (containing useful information as well) would be discarded. To circumvent this, and maintain the overall integrity of the matrix, we performed Winsorization^60^, where we set boundaries of the extreme values to the values of the 5 and 95%th quartiles, respectively.

To compare the chips, individual genes are converted into *z*-scores by subtracting the mean from the log2exp, and division by the s.d.

Arrays are compared on a per-donor basis (Donor_i unstimulated control, IFNγ-stimulated, where *i*=1,2,3). For each pair, the arrays are merged into a combined matrix. We calculate the Manhattan distance^61^ between all cells based on their gene parameters, and represent the clustering using the minimum spanning tree^62^.

### Statistical analysis

Data are given as mean ±s.d. Moreover, ‘*n*' indicates the number of independent experiments or number of animals/samples. Tests with a *P* value less than 0.05 were considered statistically significant. Pairwise group comparisons were performed using a Student's *t-*test (GraphPad prism 5, Prism Software Inc. (La Jolla, CA)). If F test showed the variance was significantly different, unpaired *t*-test with Welch's correction was performed. Exclusion criteria were set by Grubbs' test. The experiments were not randomized. No statistical method was used to predetermine sample size. The experiments were not randomized.

### Data availability

The data that support the findings of this study are available within the article and its Supplementary Information Files.

## Additional information

**How to cite this article**: Iwata, H. *et al*. PARP9 and PARP14 cross-regulate macrophage activation via STAT1 ADP-ribosylation. *Nat. Commun.* **7,** 12849 doi: 10.1038/ncomms12849 (2016).

## Supplementary Material

Supplementary Information Supplementary Figures 1-20 and Supplementary Tables 1-4.
