# Supplementary material for: PARP9 and PARP14 cross-regulate macrophage activation via STAT1 ADP-ribosylation
Source: Nat Commun. 2016 Oct 31;7:12849. doi: 10.1038/ncomms12849 (PMC5095532; doi:10.1038/ncomms12849)
Supplement: Supplementary Information — Supplementary Figures 1-20 and Supplementary Tables 1-4. [file ncomms12849-s1.pdf]

Supplementary Figure 1

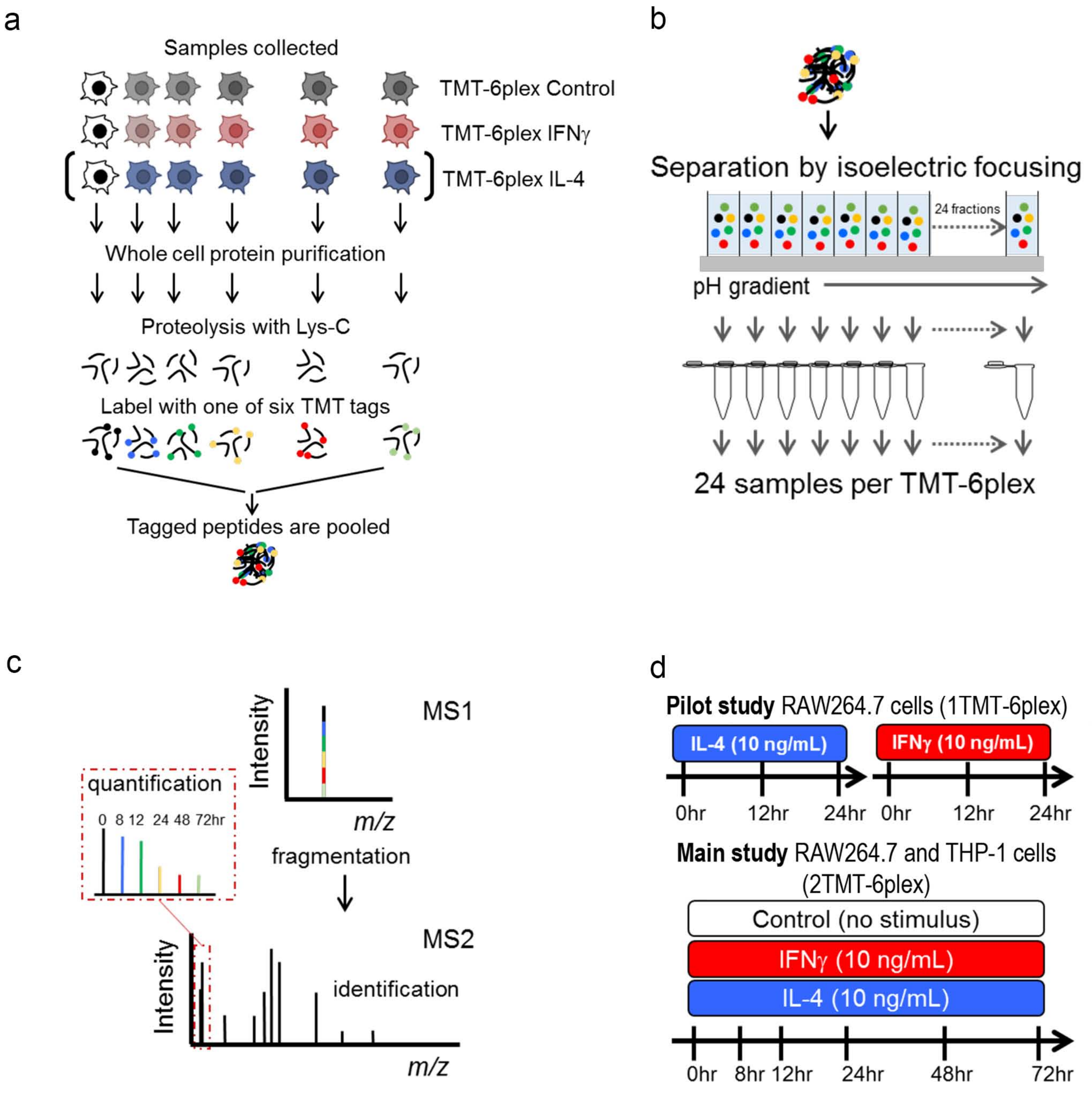

Supplementary Figure 1: Tandem mass tagging (TMT)-6plex global proteomics dataset analyses. (**a**) Overview of the TMT strategy that labels six time points, for example, in the IL-4 stimulation experiment for mass spectrometric analysis. Whole cell lysate proteomes were in-solution proteolyzed with Lys-C endopeptidase and labeled with one of six TMT-6plex reporter tags (colored circles). The labeled peptides were then pooled for isoelectric focusing into 24 fractions (**b**) subsequent mass spectrometric analysis of each fraction. (**c**) Each fraction was then analyzed by LC-MS/MS for simultaneous identification and quantification of the peptides. (**d**) A summary of the TMT-6plex experimental designs presented in this study.

Supplementary Figure 2

Pilot study RAW264.7 cells (n = 3017 proteins)

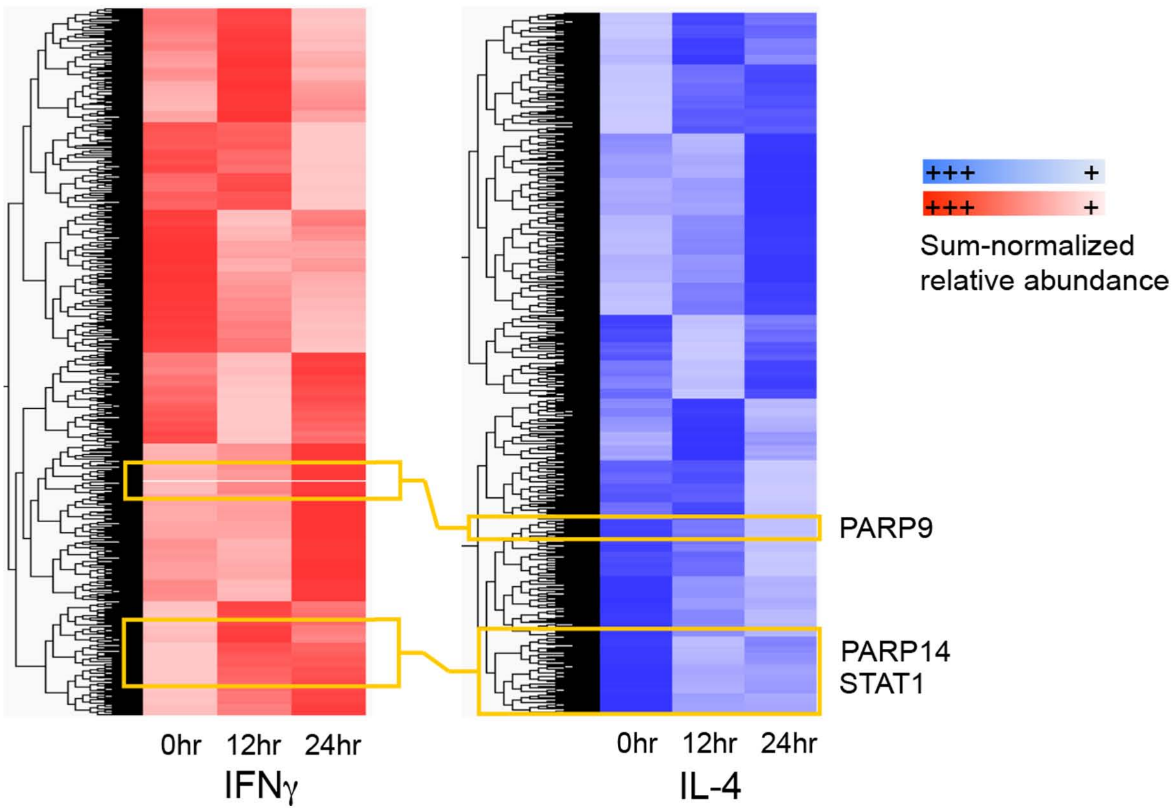

Supplementary Figure 2: Hierarchical analysis performed on the sum-normalized protein abundances from the pilot study outlined in Supplementary Fig. 1d. STAT1 was contained within clusters whose abundances increased (IFNγ) and decreased (IL-4) over the stimulation periods. PARP14 clustered with STAT1 in each experiment. PARP9 was located in clusters with similar trends as PARP14 and STAT1. Hierarchical clustering was performed using Qlucore Software (www.Qlucore.com)

Supplementary Figure 3

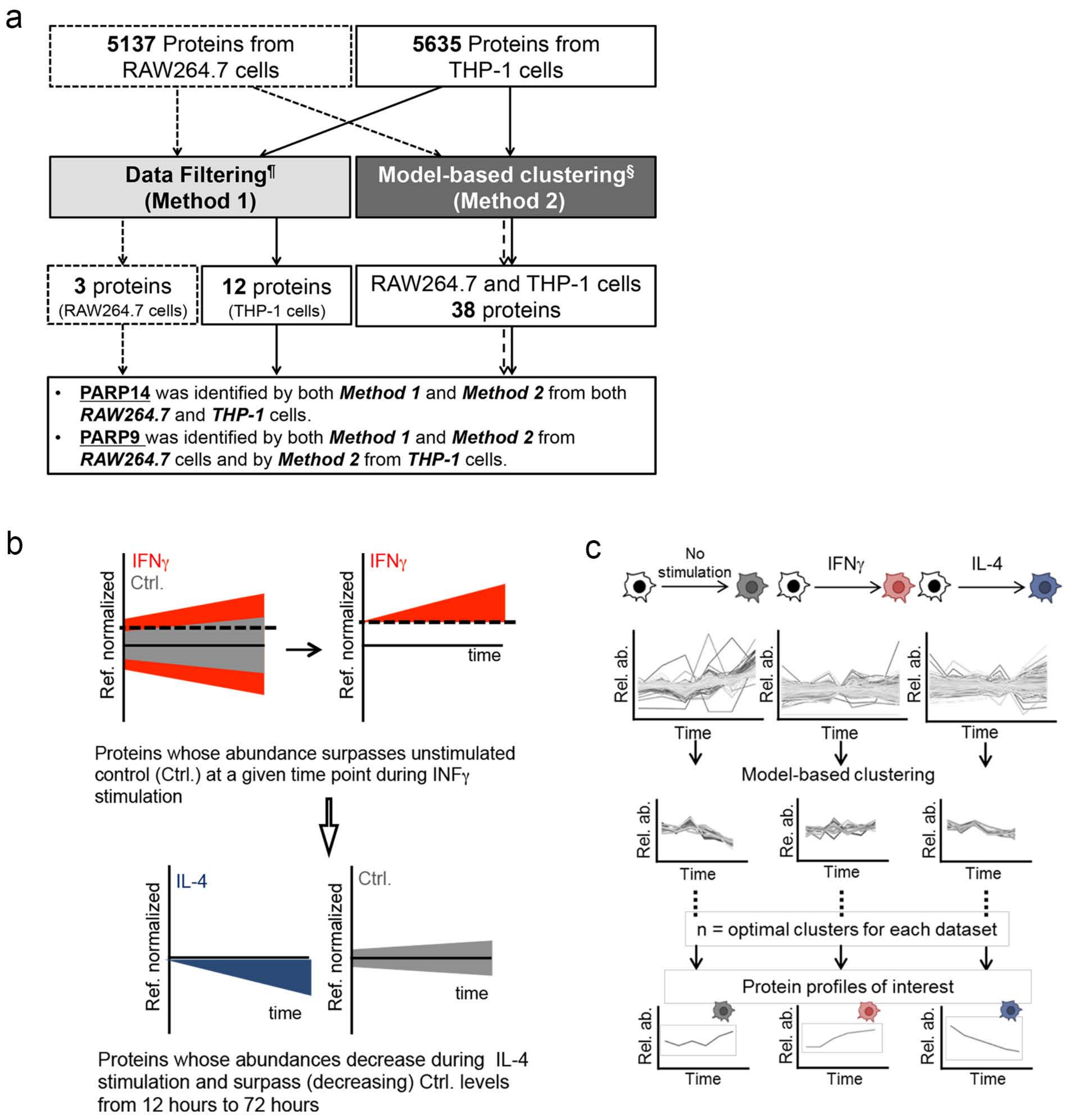

Supplementary Figure 3: A scheme for two bioinformatics methods to identify regulator protein(s) of macrophage activation. (**a**) Summary of the input and outputs of the data filtering and model-based clustering bioinformatics strategies. **(b)** Outline of the data filtering strategy that used the unstimulated control M(-) to find proteins increasing in M(IFNγ) and decreasing in M(IL-4). The maximum magnitude in fold-change (positive or negative direction) of the M(-) (indicated by gray area) represents the magnitude of fluctuation in signal. First, using the dataset in IFNγ stimulation (indicated by red area) against the M(-) for RAW264.7 cells, we chose a 1.34-fold cut-off since the magnitude in M(-) was maximal at this value at 8 hours. In addition, IFNγ had clearly induced signal beyond the cut-off. Second, we extracted all proteins that met this criterion (n = 37). We then cross referenced the proteins in M(IL-4) (indicated by blue area) and filtered out proteins whose profiles decreased overtime. We then limited the proteins to those whose profiles were also accounted for in unstimulated control. Only three proteins, including PARP14, met these criteria. We used the same cut-off for the THP-1 cells, although THP-1 cells had a larger magnitude in M(-) fluctuation; however, the same filtering procedure only produced 12 proteins, including STAT1, PARP14 and PARP9, as final candidates. **(c)** Model-based clustering of proteins accounts for the variance within each dataset, and then assumes that each protein abundance profile is derived from a mixture of underlying populations, each corresponding to a group or cluster We examined the clusters in each dataset and searched for clusters whose abundances increased over time in M(IFNγ) and decreased over time in M(IL-4), but whose abundances did not change over time in M(-).

Supplementary Figure 4

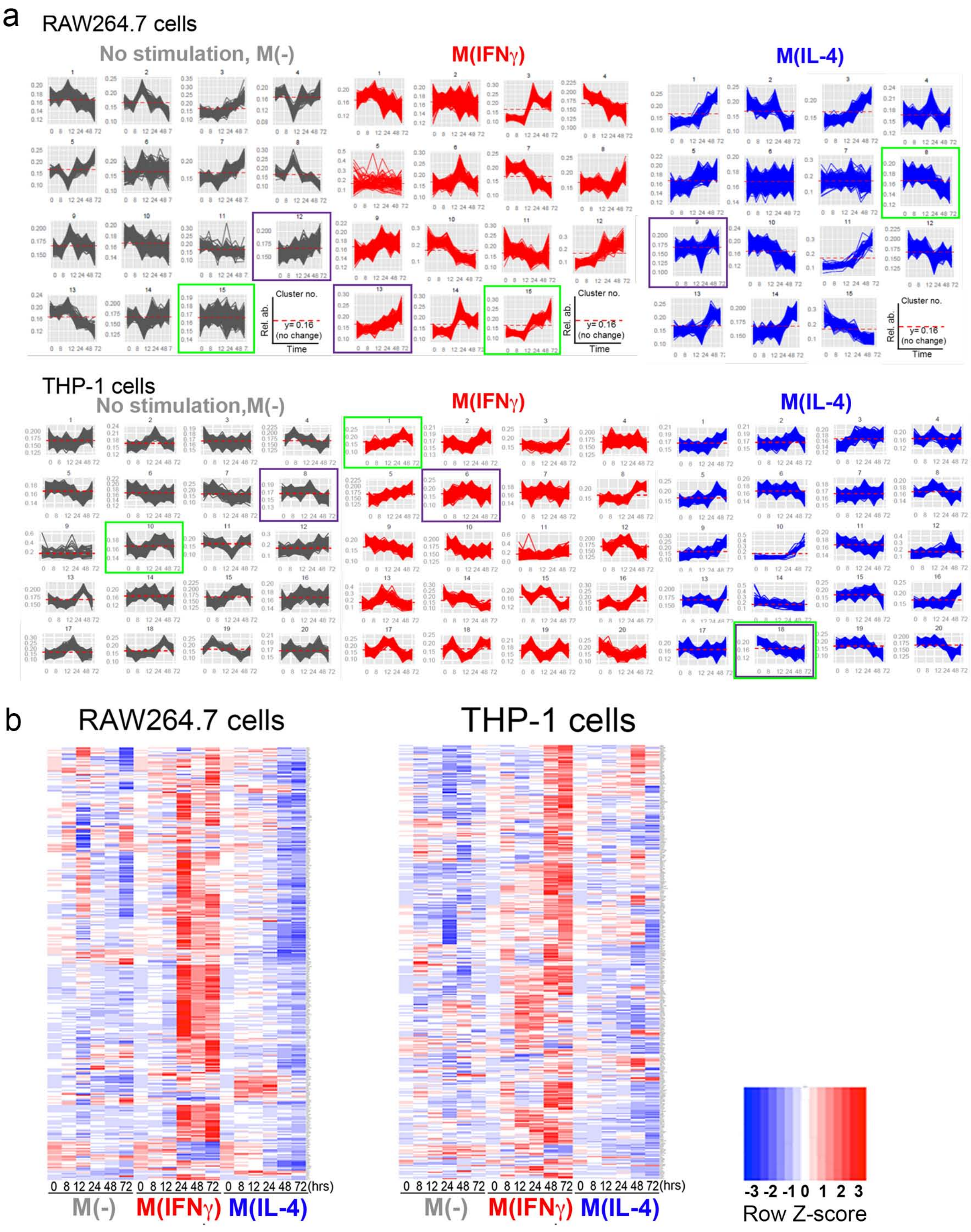

Supplementary Figure 4: Model-based clustering output for RAW264.7 and THP-1 TMT-6plex proteomics studies. M(-), grey; M(IFNγ), red; M(IL-4) blue traces. y-axis - the sum normalized relative abundance; x-axis – the time points after stimulation collected for TMT analysis. The dashed red line indicates the y = 0.16 threshold (i.e., sum-normalized no change). The location of PARP14 and PARP9 are indicated. **(b)** Hierarchical clustering of 490 and 414 proteins from identified in datasets of RAW264.7 cells and THP-1 cells, respectively. List of proteins are shown in the **Supplementary Table 3**.

Supplementary Figure 5

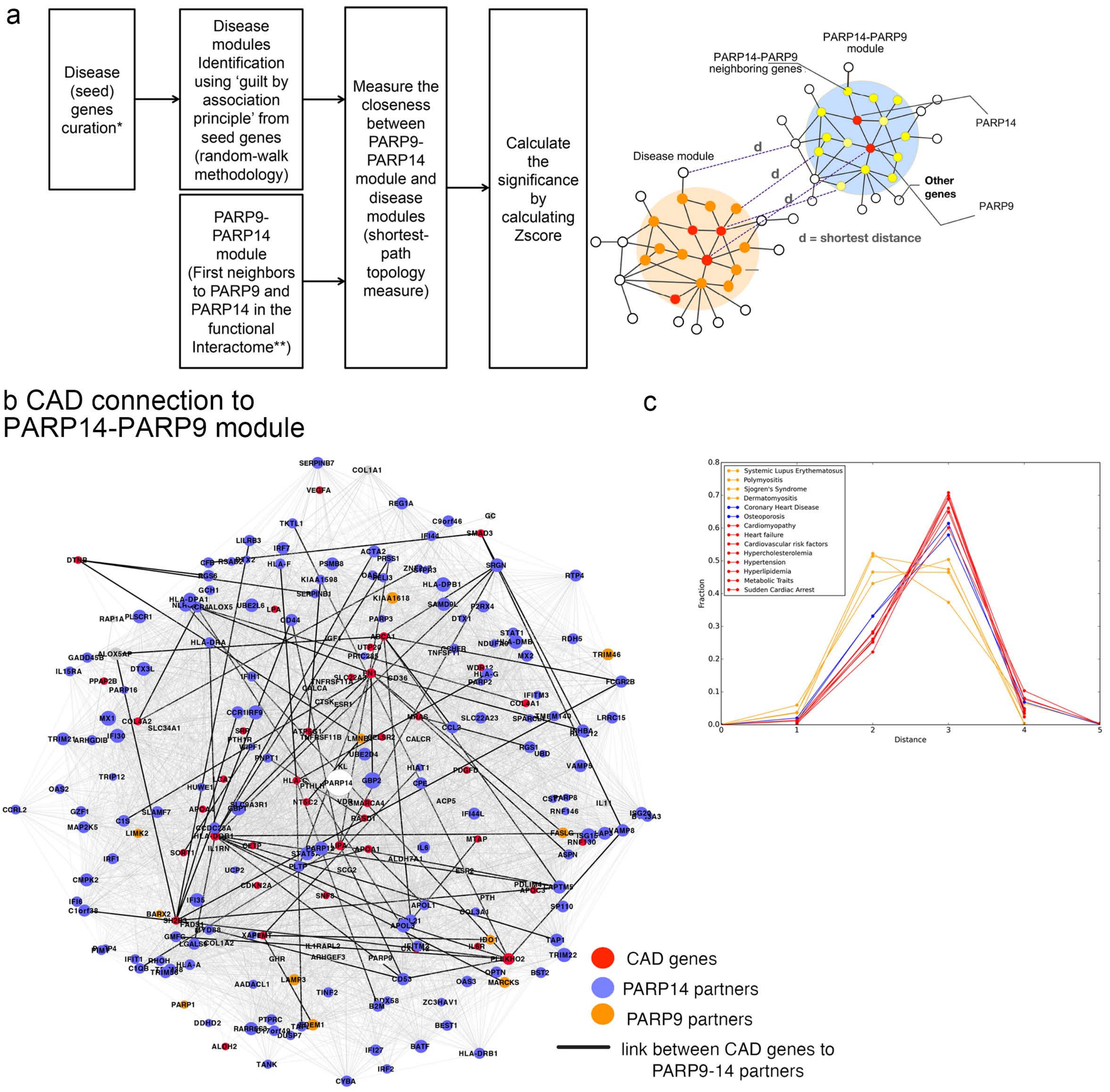

Supplementary Figure 5: Closeness between PARP9 and PARP14 module and disease modules (a) A workflow of network analysis to measure the closeness between the PARP9-PARP14 module (blue circle) and an example disease module (orange circle). **(b)** Connection of the PARP9-PARP14 module to CAD genes in the interactome. **(c)** Distribution of shortest distances between each disease module and the PARP9-PARP14 module. *: coronary artery diseases (CAD), osteoporosis, polymyositis (PM), dermatomyositis (DM), systemic lupus erythematous (SLE), metabolic traits, cardiovascular risk factor, hyperlipidemia, hyperchlestrolemia, hypertension, cardiomyopathy and sudden cardiac arrest **: GWAS, OMIM and MalaCards

Supplementary Figure 6

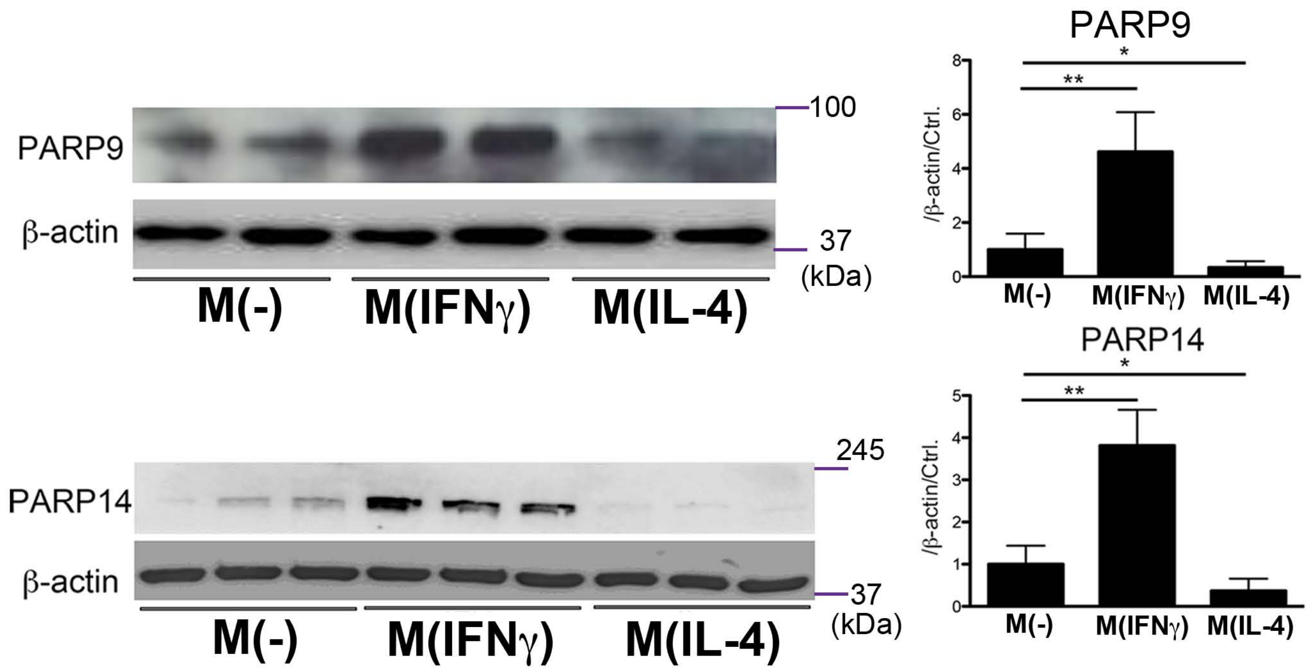

Supplementary Figure 6: PARP9 and PARP14 protein expression in M(-), M(IFNγ) and M(IL-4). Western blotting of PARP9 and PARP14 protein expression in THP-1 cells under stimulation for 24 hours (left panels), and their quantification (right panels) (n = 4).

Supplementary Figure 7

a Normal carotid artery, PARP9, PARP14 and CD68

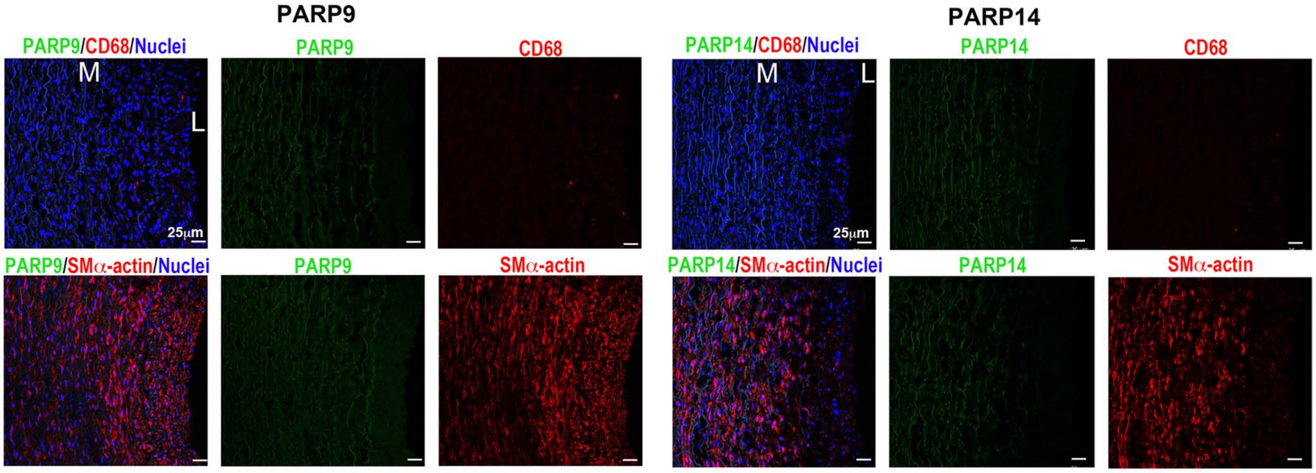

b Carotid artery plaque, PARP9, PARP14 and SMα-actin/ CD68/ CD31

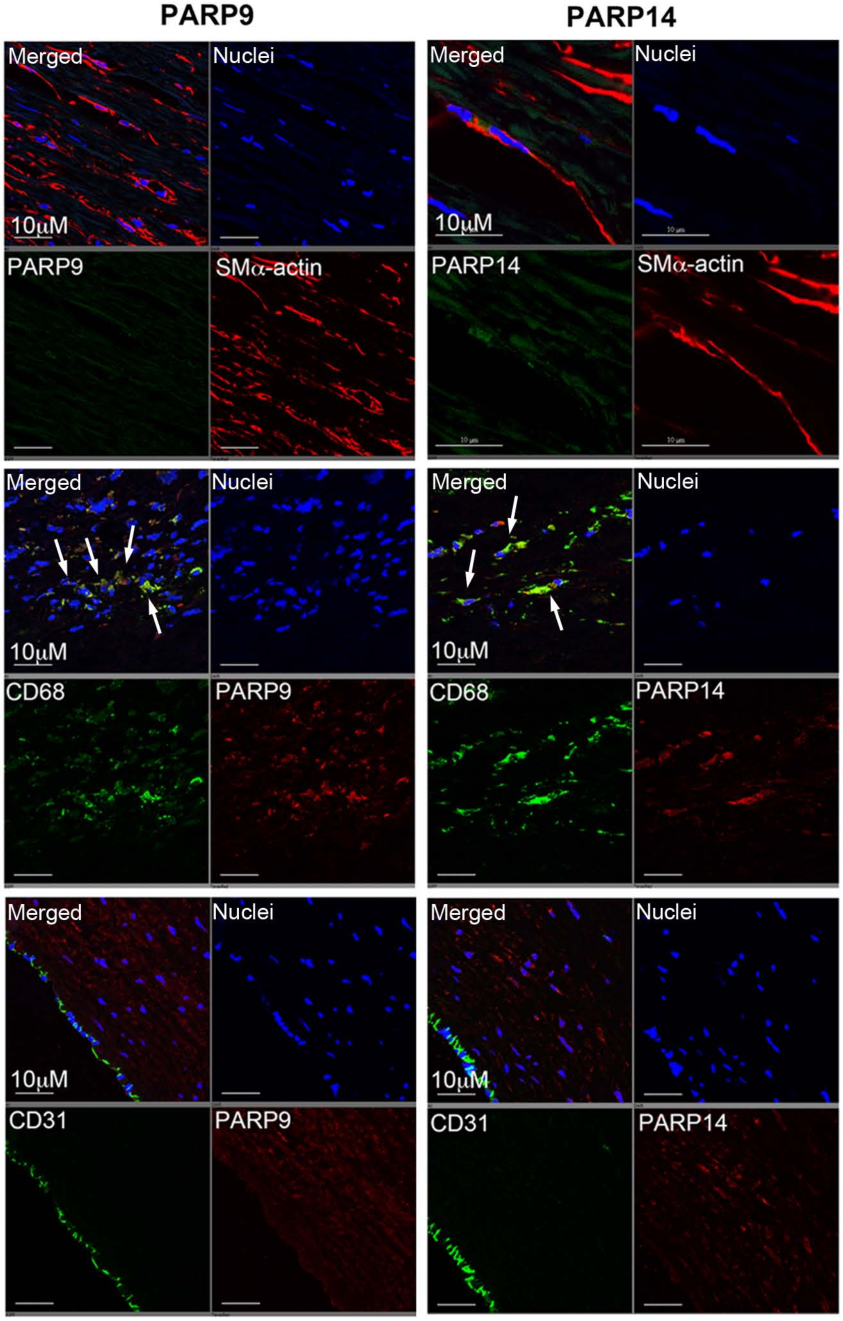

Supplementary Figure 7: PARP9 and PARP14 expression in non-diseased and diseased carotid arteries (a) PARP9 and PARP14 expression in carotid arteries without no apparent atherosclerotic changes in combination with CD68. Scale bars indicate 25μm. **(b)** PARP9 and PARP14 expression in atherosclerotic carotid arteries in combination with SMα-actin, CD31 and CD68. Scale bars: 10 μm. Arrows indicate co-localized signal.

Supplementary Figure 8

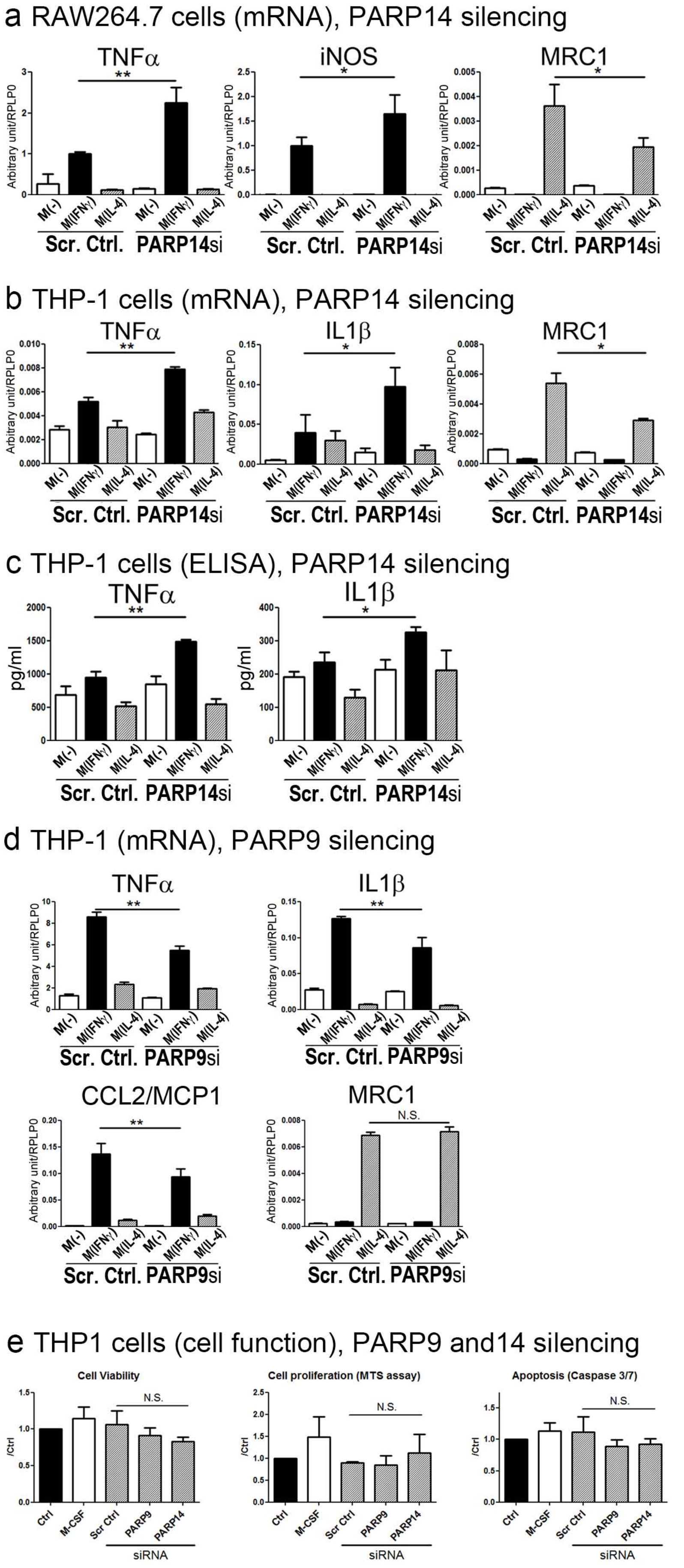

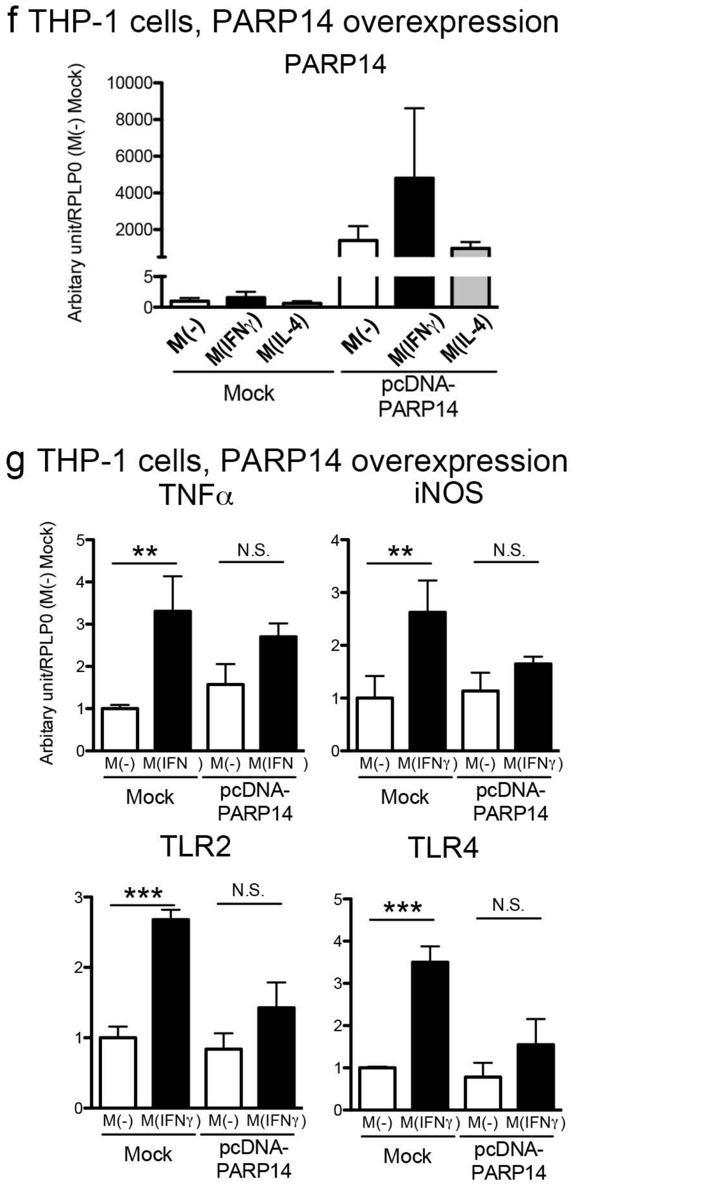

Supplementary Figure 8: The molecular functions of PARP14 and PARP9 in macrophage activation *in vitro*. (**a, b**) The consequence of PARP14 silencing on IFNγ pathway (TNFα, iNOS and IL-1β) and IL-4 pathway (MRC1) gene expression (n = 3) in mouse RAW264.7 and human THP-1 cells. **(c)** PARP14 silencing increased expression levels of TNFα and IL-1β proteins in the media of THP-1 cells (n = 3). **(d)** The consequence of PARP9 silencing on IFNγ pathway (TNFα, IL-1β and CCL2/MCP1) and IL-4 pathway (MRC1) gene expression in THP-1 cells (n = 3). (**e**) PARP14 and PARP 9 silencing had no significant effects on viability, proliferation and apoptosis of mouse bone marrow-derived macrophages (n = 3). **(f and g)** Enforced expression of PARP14 in THP-1 cells increased the expression of pro-inflammatory genes TNFα, iNOS, TLR2, and TLR4 in M(IFNγ).

Supplementary Figure 9

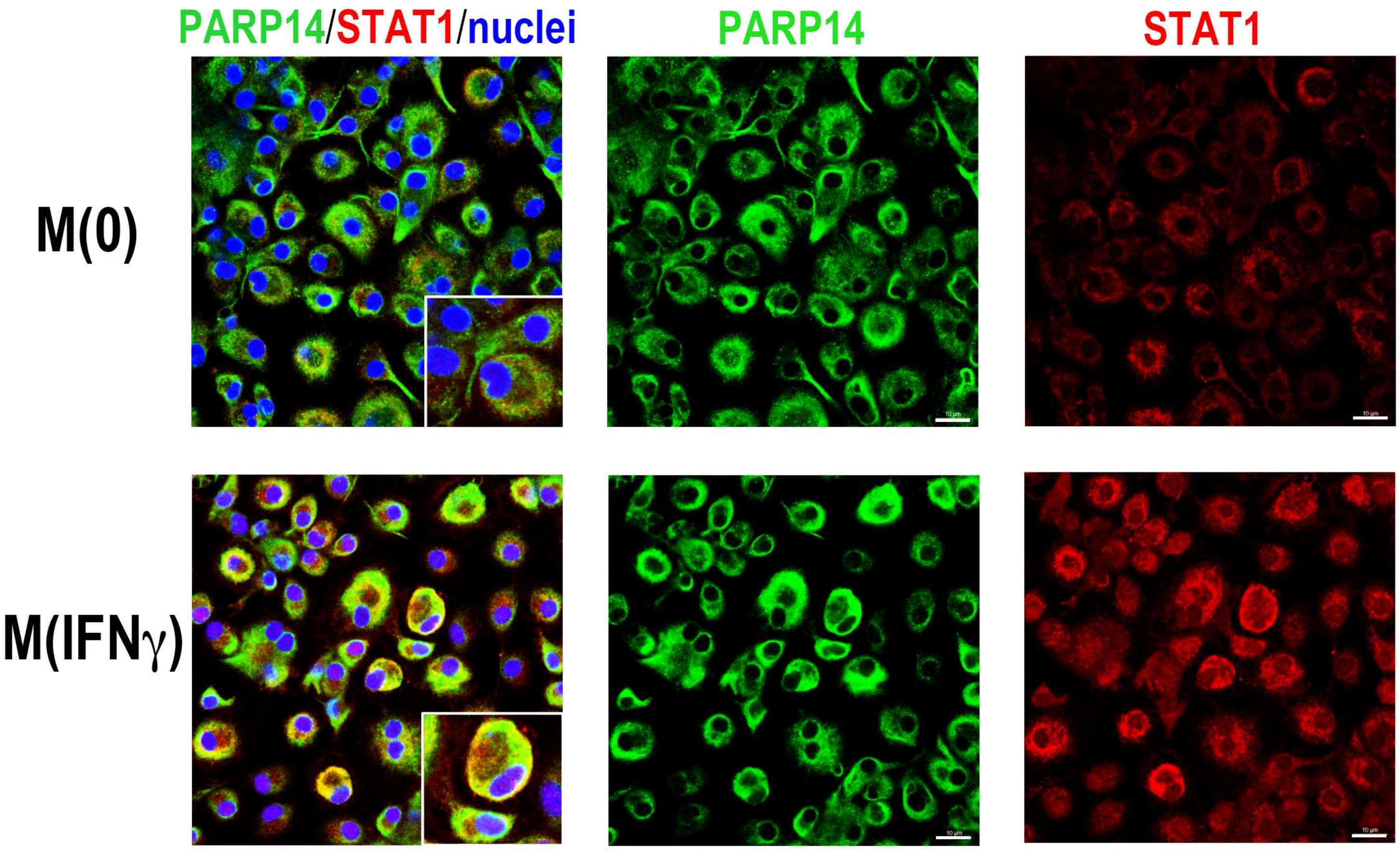

Supplementary Figure 9: Colocalization of PARP14 with STAT1 in unstimulated, M(-), and IFNγ-stimulated M(IFNγ) THP-1 cells. PARP14 (Alexa 488, green) further co-localized with STAT1 (Alexa-594, red) in the cytosol under IFNγ stimulation rather than unstimulated control. Scale bars indicate 10μm.

Supplementary Figure 10

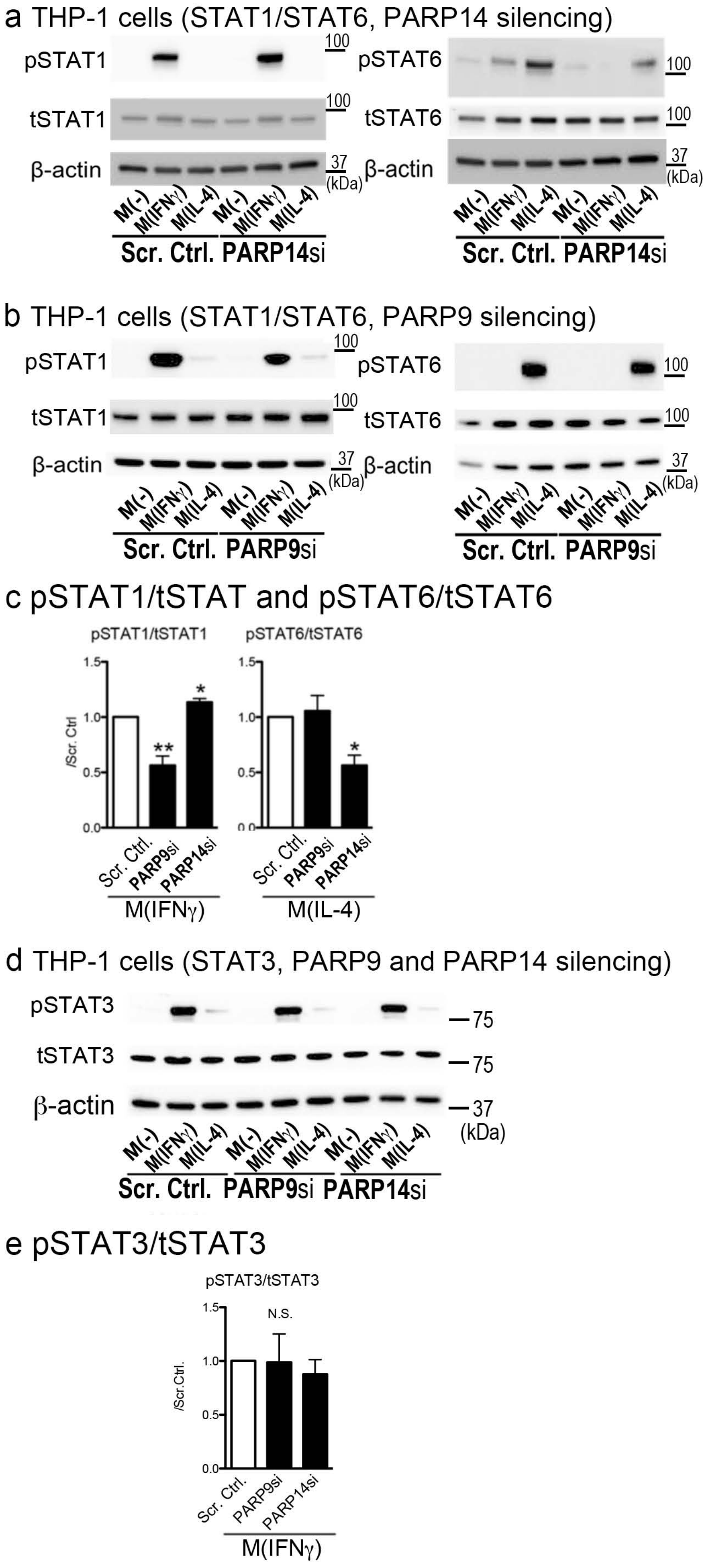

Supplementary Figure 10: The effects of PARP14 and PARP9 silencing on STAT1, 6 and 3 phosphorylation. (a) PARP14 silencing increased phosphorylation of STAT1 (pSTAT1) in IFNγ-stimulated THP-1 cells and decreased the phosphorylation of STAT6 (pSTAT6) in IL-4-stimulated THP-1 cells. **(b)** PARP9 silencing decreased phosphorylation of STAT1 in IFNγ -stimulated THP-1 cells, but had no effect on STAT6 in cells stimulated with IL-4 (THP-1). **(c)** Quantified ratios of phospho/ total STAT1 and STAT6 in PARP9 and PARP14 silencing. (n = 3) in M(IFNγ) and M(IL-4) **(d)** Silencing PARP14 or PARP9 had no effect on phosphorylation of STAT3 (pSTAT3) in THP-1 cells. **(e)** Quantification of pSTAT3/tSTAT3 in M(-), M(IFNγ) and M(IL-4).

Supplementary Figure 11

a $_{620}$KDEAFR**SHYKPEQMGK**DGRG$_{640}$

b

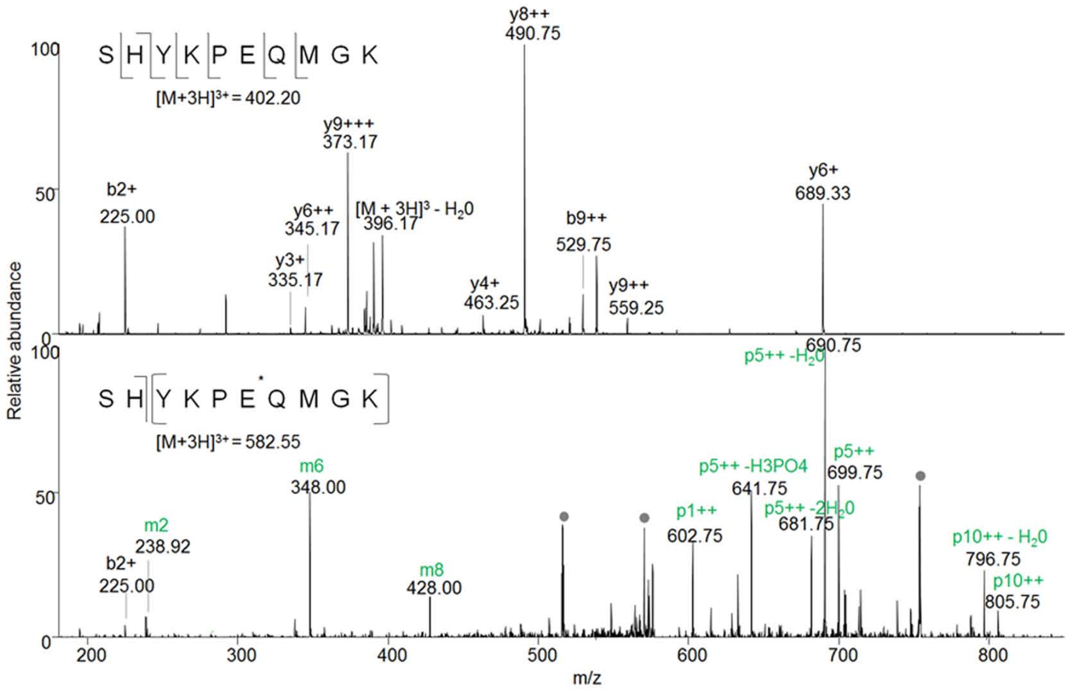

Supplementary Figure 11: Mass spectrometric-based detection of ribosylated STAT6. (a) A part of amino acid sequence of human STAT6. Green amino acids indicate the ribosylated peptide; the likely but not confirmed ribosylation site is underlined. STAT6 is known to be phosphorylated at indicated tyrosine (red). **(b)** MS/MS spectra for the mono-ADP-ribosylated peptide and the corresponding unmodified form. ADP-ribose fragments are annotated in green. *, likely ribosylation site; [ ], ribosylation moiety is contained within these amino acids, m, oxidized Met. The grey circles indicate background or undetermined ions. **(c)** Reference for the annotation of ADP-ribose fragment ions.

Supplementary Figure 12

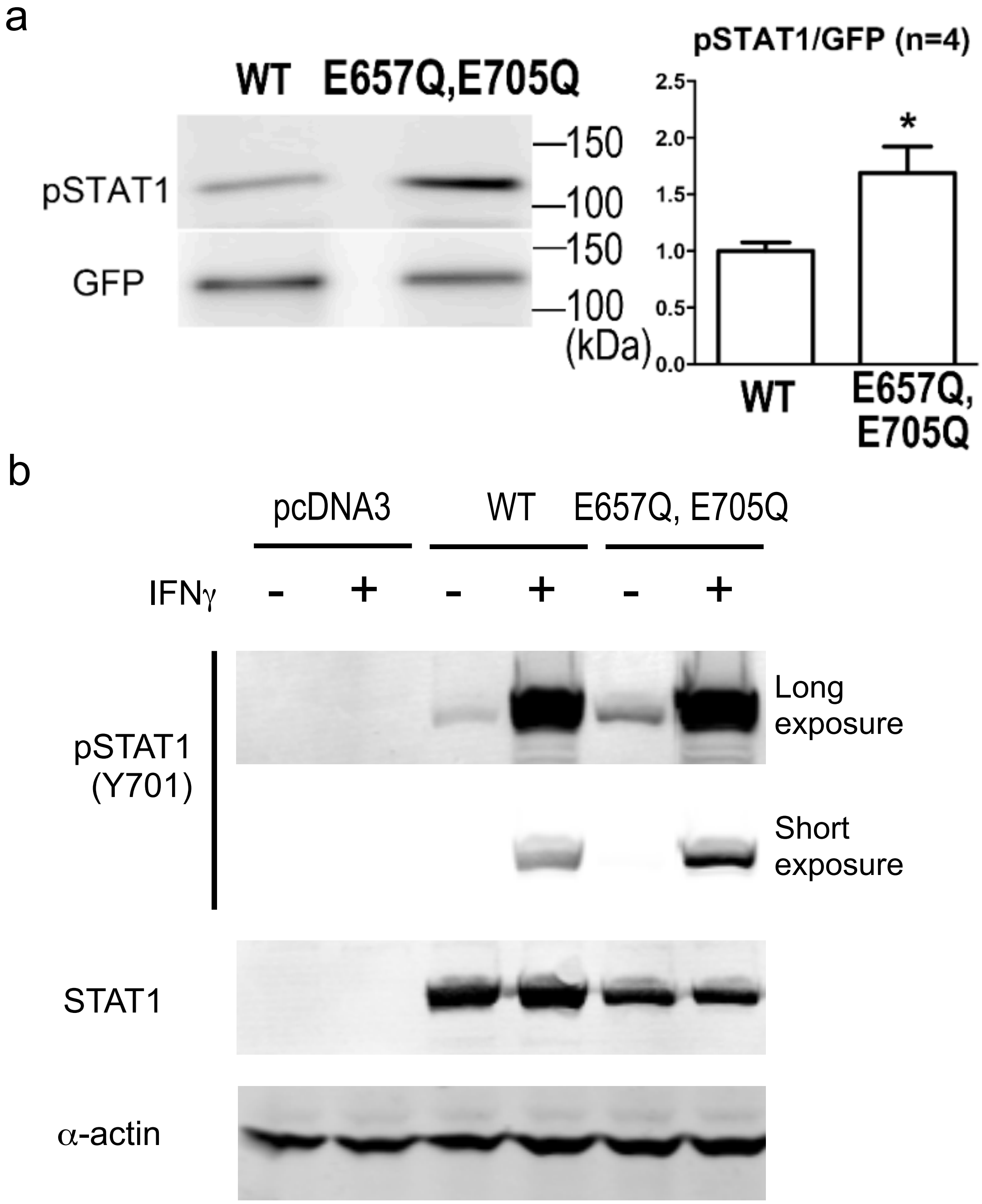

Supplementary Figure 12: The effects of wild type and mutated STAT1 on phosphorylation of STAT1 (Tyr701) HEK293 cells were transfected with wild type (WT) or mutated STAT1 (E657Q, E705Q). Two independent experiments at the Masanori Aikawa (a) and Mark Boothby (b) laboratories demonstrated similar results. * indicates $p < 0.05$ by Student's t-test

Supplementary Figure 13

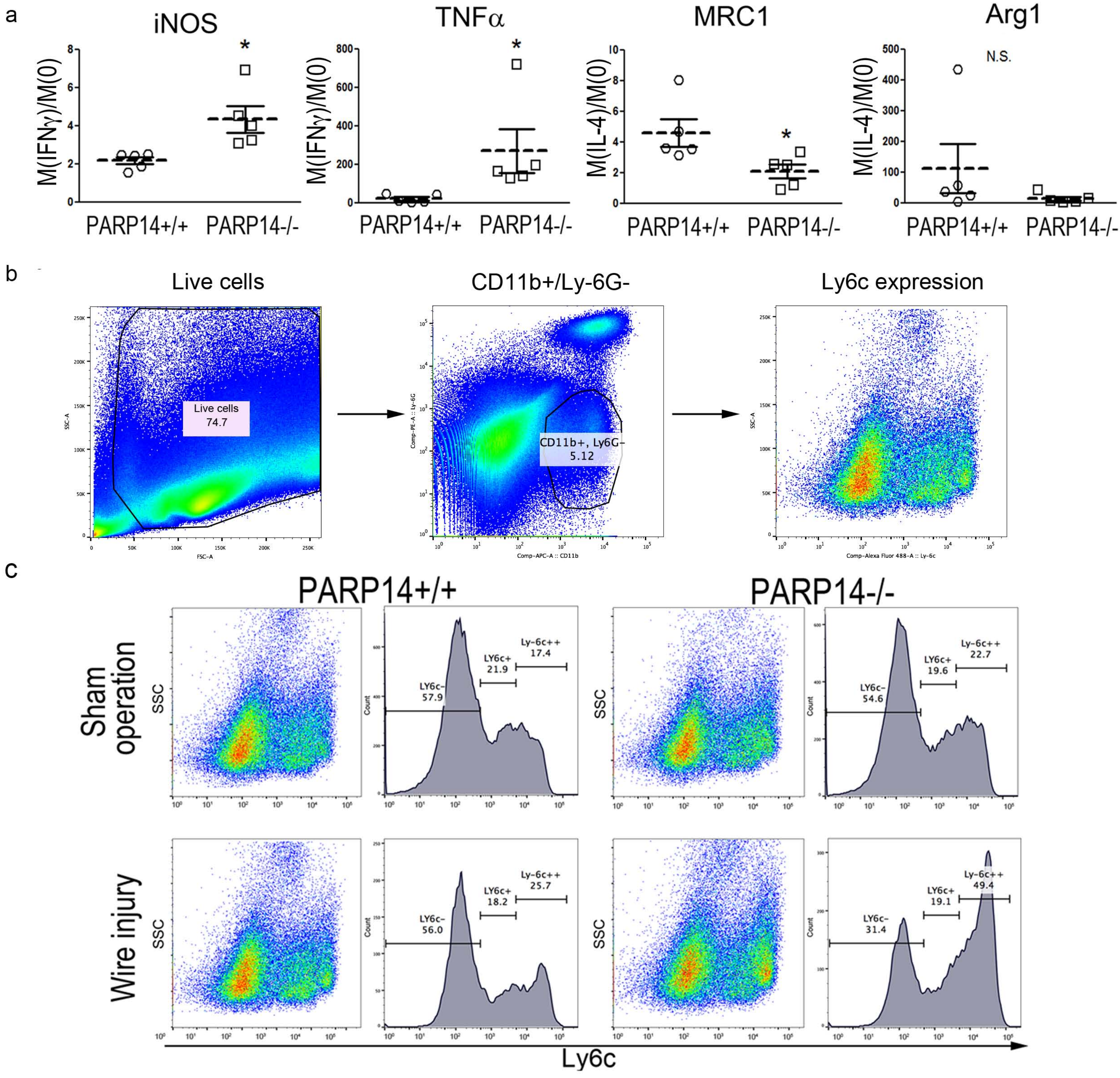

Supplementary Figure 13: Characteristics of bone marrow-derived macrophages in PARP14-/- and +/+ mice, and splenic inflammatory monocytes/macrophages (a) IFNγ - and IL-4-pathway gene expression data from bone marrow derived macrophages from PARP14$^{-/-}$ and PARP14$^{+/+}$ mice. Each data point is the average of quadruplicate samples per donor (n = 5). * and N.S. indicate p < 0.05 and not significant by Student's t-test. **(b)** Ly6c expression in splenic CD11b+/LY6g- monocytes/macrophages. **(c)** Ly6c expression in splenic monocytes/macrophages in sham operated and animals after wire injury in PARP14$^{+/+}$ and PARP14$^{-/-}$ mice.

Supplementary Figure 14

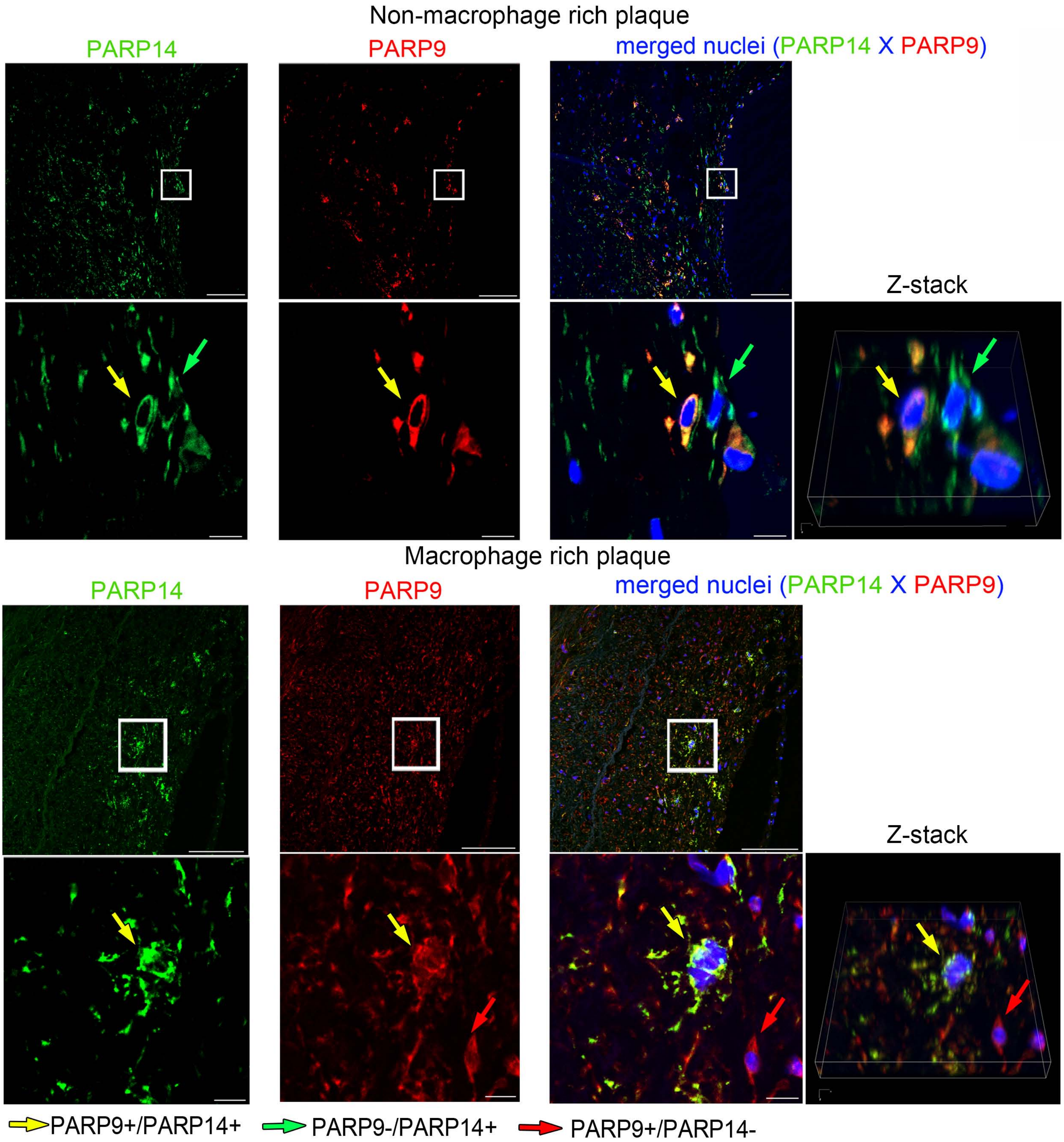

Supplementary Figure 14: Differential expression of PARP9/PARP14 in macrophage-rich and no macrophage-rich plaques. Green arrows, PARP9-/PARP14+ cells; yellow arrows, PARP9+/PARP14+ cells; red arrows: PARP9+/PARP14- cells. Scale bars indicate 10μm.

Supplementary Figure 15

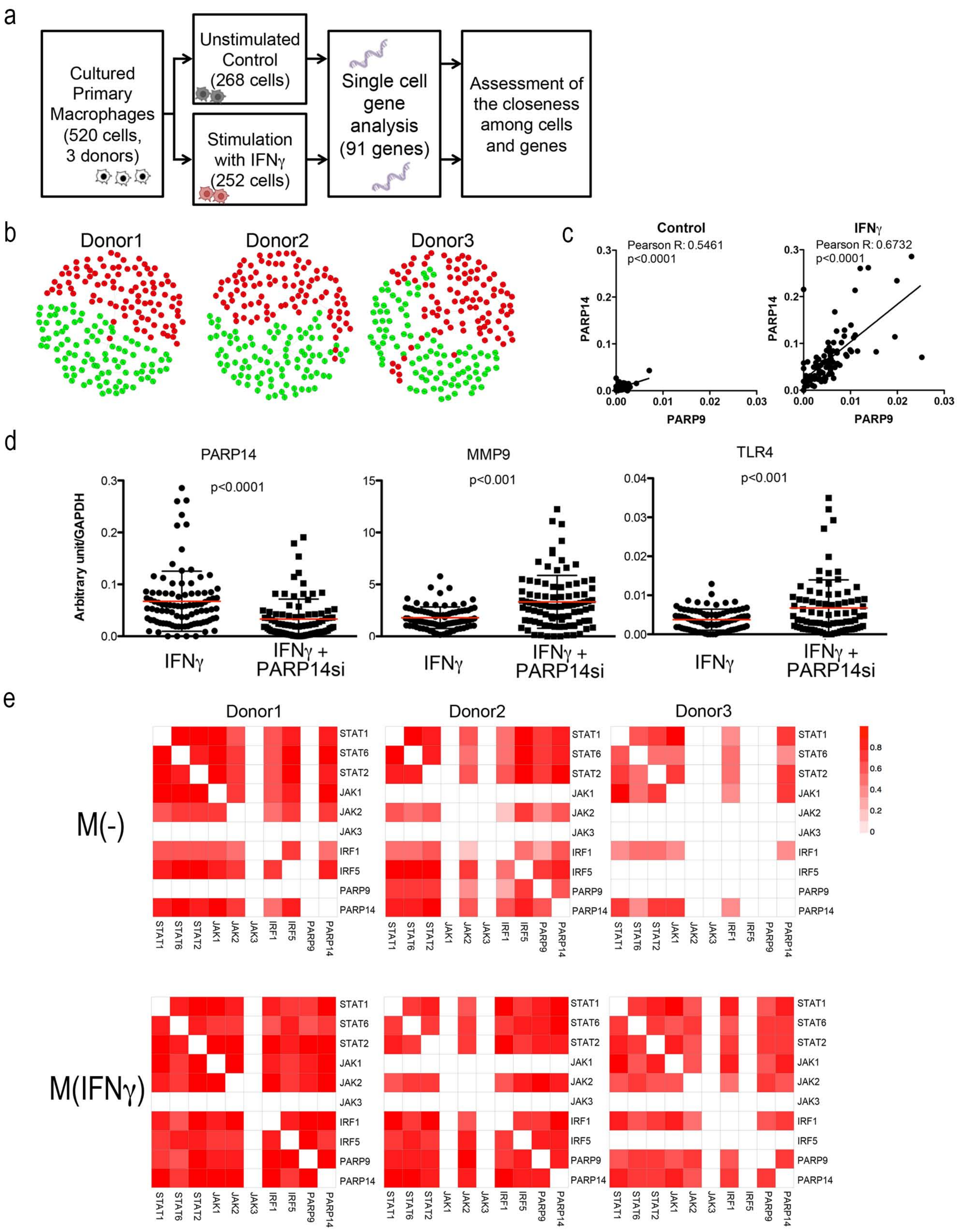

Supplementary Figure 15: Overview and additional analysis regarding heterogeneity and PARP14 function in IFNγ-induced activation of human primary macrophages. (a) Schema of the workflow in single cell gene profiling in human primary macrophages. **(b)** Heterogeneity in M(IFNγ) (red) compared to M(-) (green) in individual donor samples. **(c)** The positive correlation of PARP9 and PARP14 gene expression at the single cell level. **(d)** Single cell gene analysis in M(IFNγ) with or without PARP14 silencing. **(e)** Similarity maps of samples from all donors (Donors 1 to 3) in both M(-) and M(IFNγ), demonstrating the closeness of genes related IFNγ pathway in macrophages.

Supplementary Figure 16

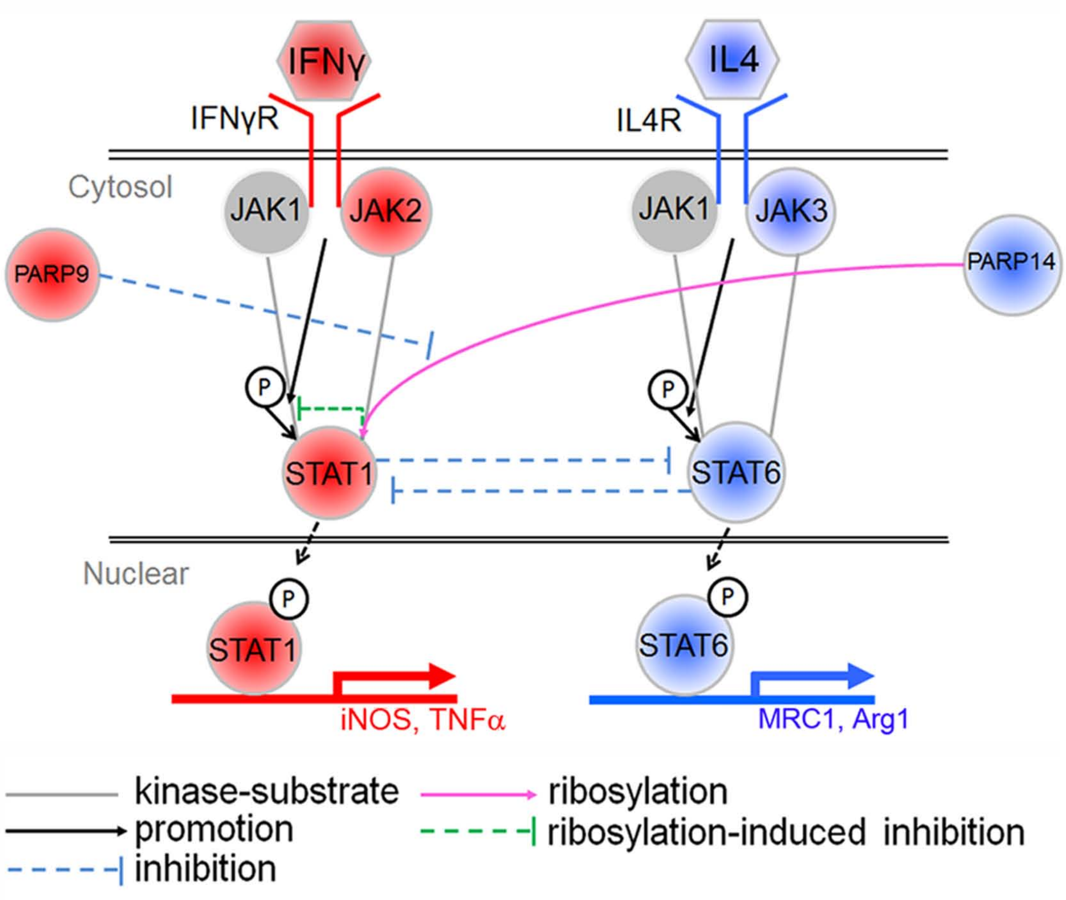

Supplementary Figure 16: A model for multidirectional macrophage activation incorporating our own novel findings on PARP14 and PARP9.

Supplementary Figure 17

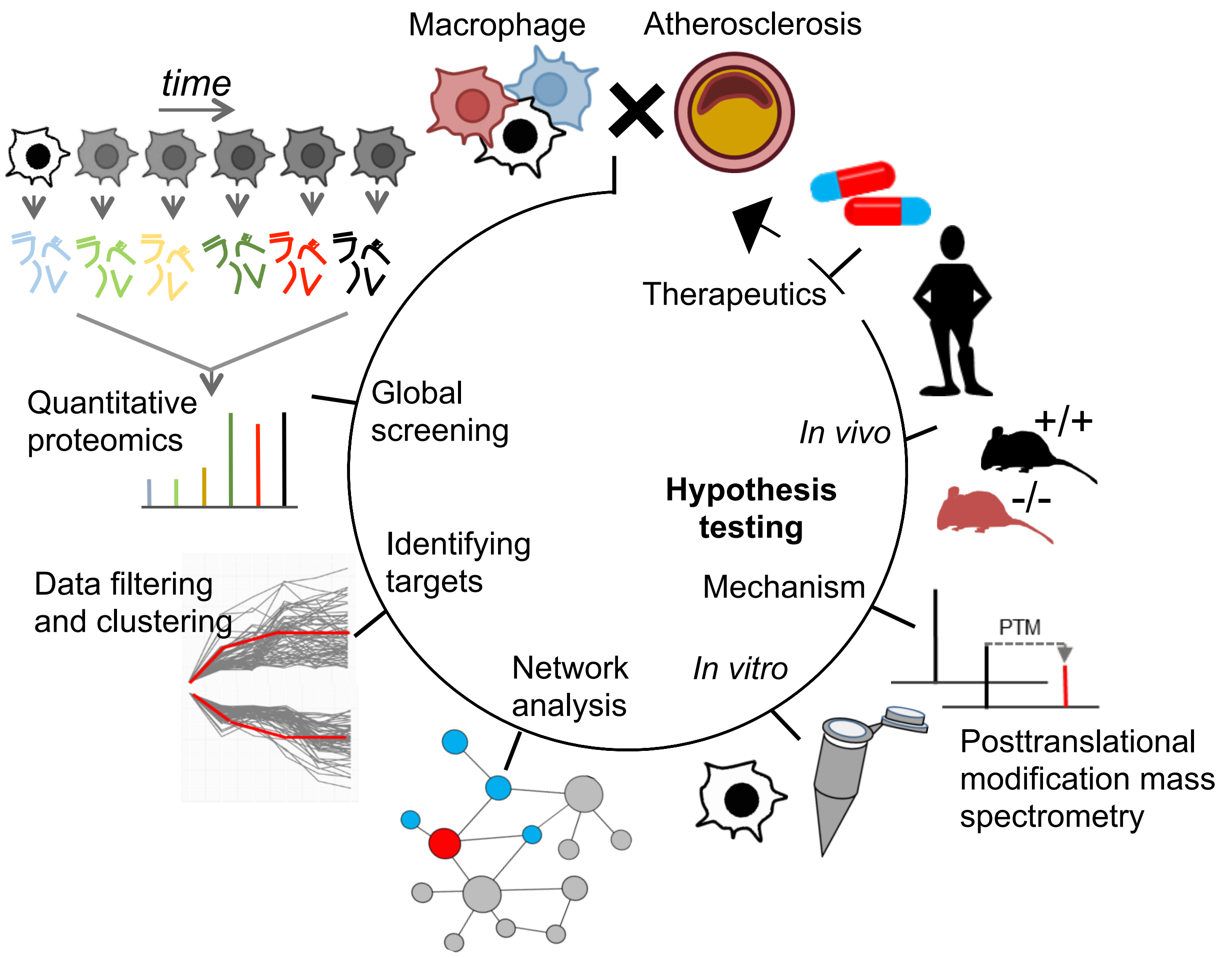

Supplementary Figure 17: Schematic workflow of integrated target discovery research from global screening to validations to drug development.

Supplementary Figure 18

Figure 3c

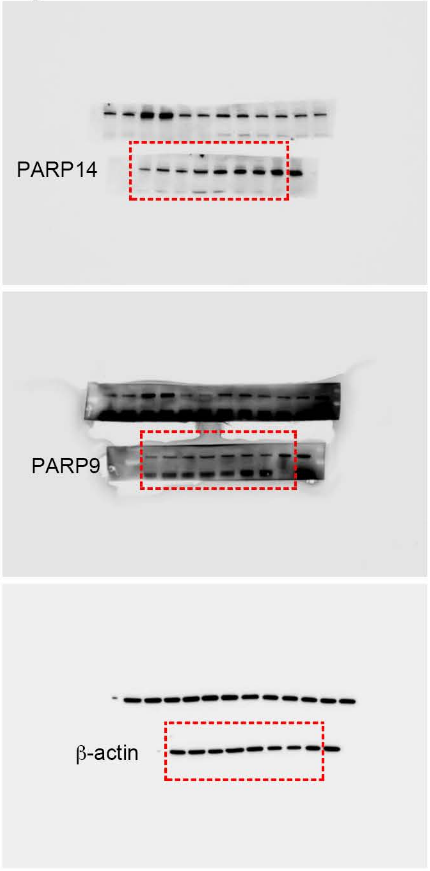

Figure 5d

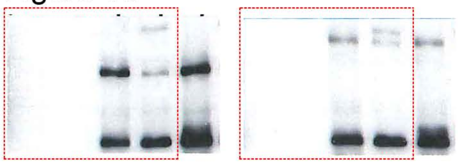

Figure 6c

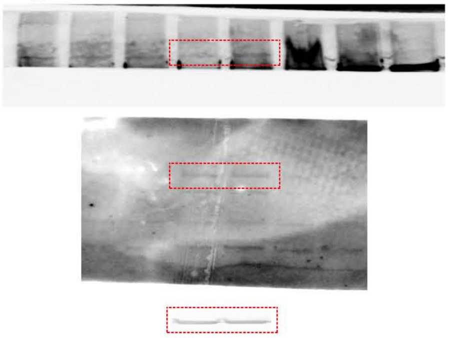

Figure 7c

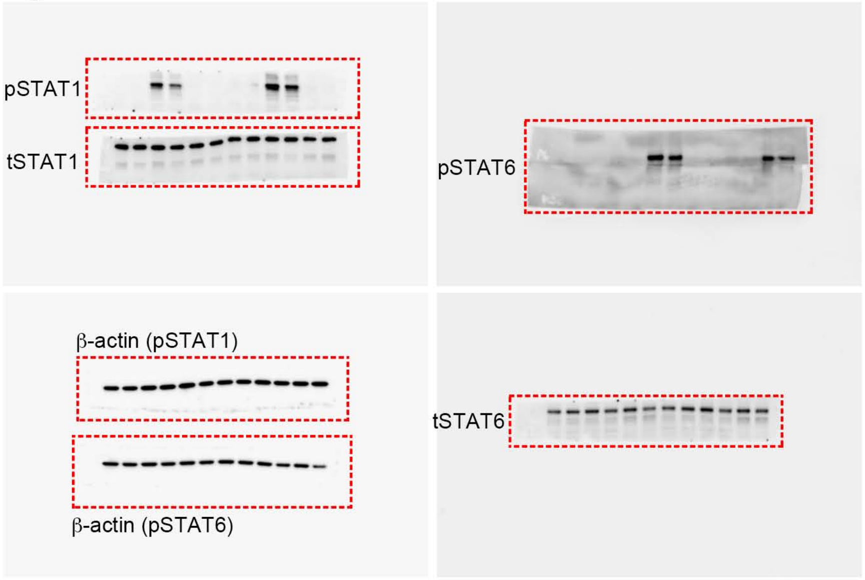

Supplementary Figure 18: Uncropped western blotting images (Figure 3c – 7c)

Supplemantary Figure 19

Figure 10a

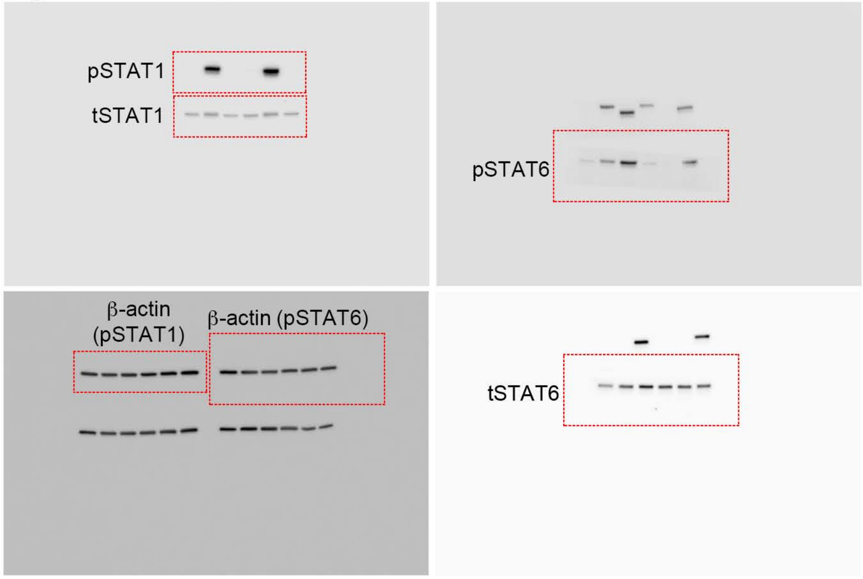

Figure 10d

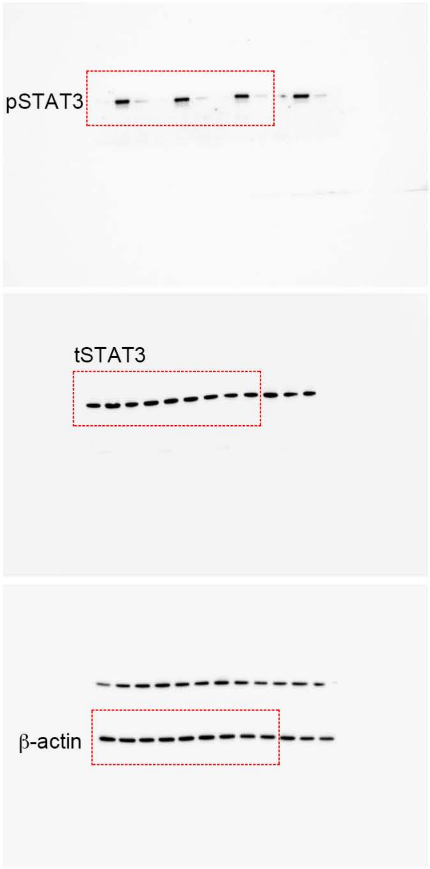

Figure 10b

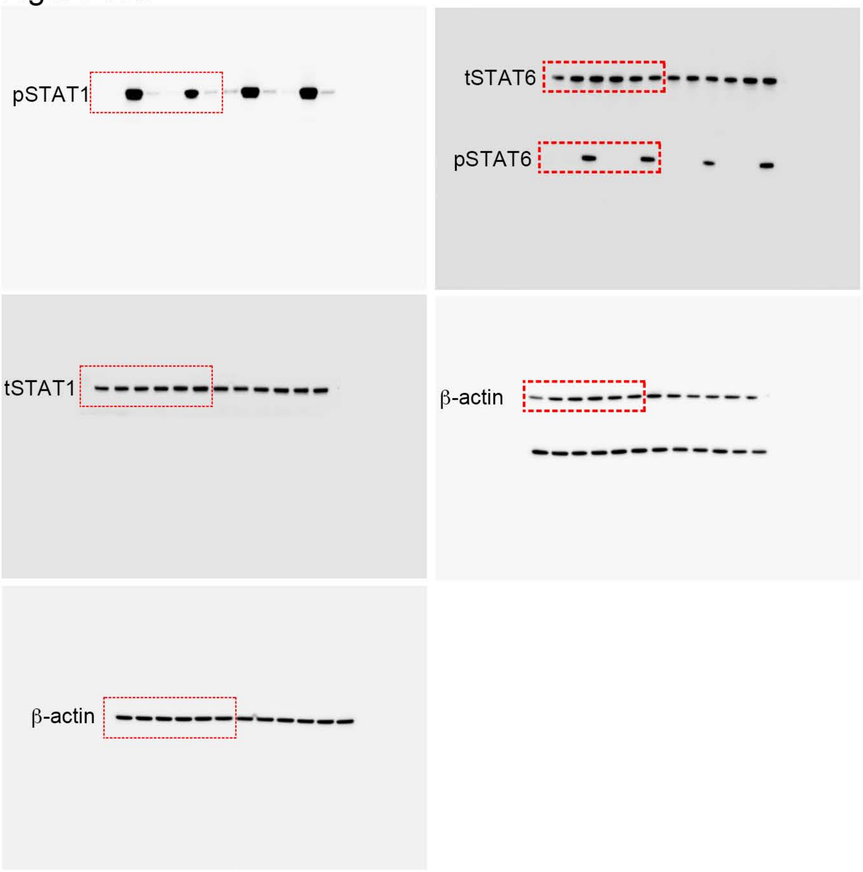

Supplementary Figure 19: Uncropped western blotting images (Figure 10a, b and d)

Supplementary Figure 20

Supplementary Figure 6

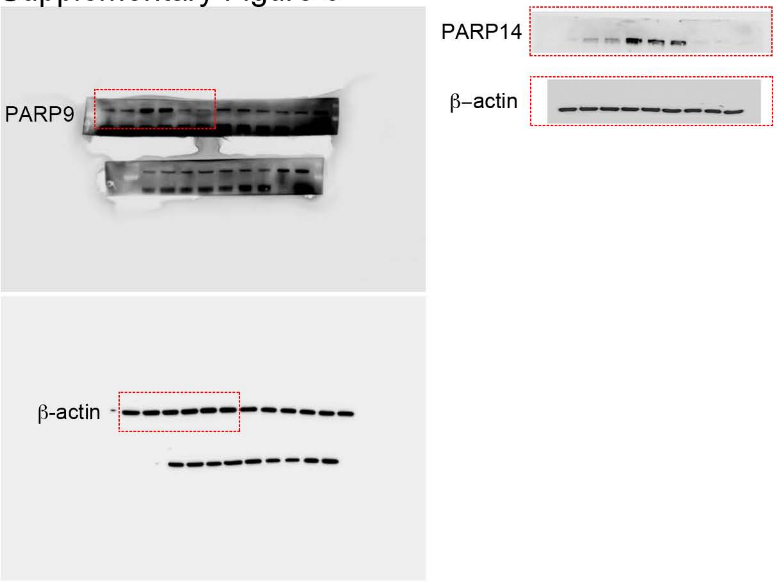

Supplementary Figure 12a

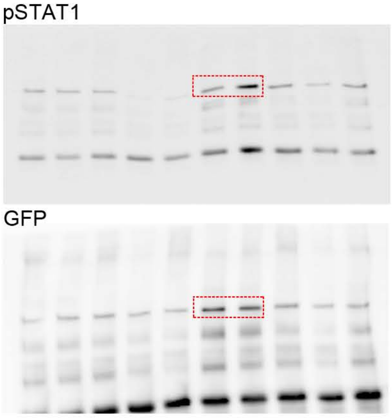

Supplementary Figure 20: Uncropped western blotting images (Supplementary Figure 6 and 12a

Supplementary Table 1: Overview of quantified proteomes

Total proteins quantified by MS/MS					
**RAW264.7 cells**			**THP-1 cells**		
Untimulated Control	IFNγ	IL-4	Untimulated control	IFNγ	IL-4
4,234	6,393	5,470	6,338	6,817	6,772
**Filtered for the number of unique peptides >1**					
2,934	4,786	3,966	4,713	4,623	5,027

Supplementary Table 2: The number of unique peptides and spectral counts for PARP9 and PARP14

RAW264.7 cells

	PARP14			PARP9		
	Control	IFNg	IL-4	Control	IFNg	IL-4
**Number of unique peptides**	6	40	13	2	8	4
**Spectral counts**	8	68	17	3	15	7

THP-1 cells

	PARP14			PARP9		
	Control	IFNg	IL-4	Control	IFNg	IL-4
**Number of unique peptides**	12	28	13	7	16	16
**Spectral counts**	22	54	20	7	30	20

Supplementary Table 3

Mouse proteins from RAW264.7 cells

No.	Gene	No.	Gene	No.	Gene	No.	Gene	No.	Gene	No.	Gene	No.	Gene
1	Aacs	71	Cmpk2	141	Fdps	211	Kpnb1	281	Nup210	351	Rad17	421	Srpk2
2	Abcb1b	72	Cnot10	142	Fhod1	212	Krr1	282	Nup88	352	Rad18	422	Srpr
3	Abcd1	73	Cnot3	143	Fkbp4	213	Las1l	283	Nup93	353	Rad50	423	Ssr4
4	Abce1	74	Cox5b	144	Fli1	214	Lbr	284	Nup98	354	Ranbp2	424	Stat1
5	Abcf2	75	Cpd	145	Fndc3b	215	Lcp2	285	Oas1a	355	Rbl1	425	Stim2
6	Abl2	76	Crnkl1	146	Ftsj3	216	Lonp2	286	Oas2	356	Rbm14	426	Stk16
7	Abt1	77	Ctbp1	147	Ftsjd2	217	Lrrc8d	287	Oas3	357	Rbm28	427	Stt3a
8	Acsl5	78	Ctbp2	148	Fxr1	218	Lsg1	288	Oasl1	358	Rbm34	428	Sun2
9	Adam10	79	Ctr9	149	Gars	219	Lsm2	289	Obfc2b	359	Rcl1	429	Supt16h
10	Afg3l1	80	Ctu2	150	Gbf1	220	Ltv1	290	Osbpl1a	360	Rfc3	430	Supt5h
11	Agpat6	81	Cul4b	151	Gbp7	221	Map3k2	291	Paf1	361	Rfc5	431	Suz12
12	Agps	82	Cyb5b	152	Gch1	222	Map3k7	292	Pak1ip1	362	Rgs3	432	Sympk
13	Ahctf1	83	Cyp51a1	153	Gemin4	223	Mapk8	293	Papola	363	Riok1	433	Tbl2
14	AI607873	84	D17Wsu104e	154	Gfm1	224	Marcksl1	294	Parp12	364	Rnaseh2b	434	Tbl3
15	Aim1	85	D1Bwg0212e	155	Gfpt1	225	Mark2	295	Parp14	365	Rnaset2	435	Tbrg4
16	Aim2	86	Dars	156	Glyr1	226	Mcm3ap	296	Parp9	366	Rnf2	436	Tcp1
17	Aldh18a1	87	Dars2	157	Gm4294	227	Mcm5	297	Pbrm1	367	Rnf213	437	Tdrd7
18	Aldh1l2	88	Daxx	158	Gm5745	228	Mdn1	298	Pcnt	368	Rnf220	438	Tecr
19	Alox5	89	Ddx10	159	Gmds	229	Med17	299	Pcx	369	Rpl10a	439	Tfip11
20	Anapc1	90	Ddx18	160	Grsf1	230	Mepce	300	Pdcd11	370	Rpl13	440	Thoc1
21	Anapc5	91	Ddx20	161	Gtf2h1	231	Mgat2	301	Pde2a	371	Rpl13a	441	Thoc2
22	Anapc7	92	Ddx27	162	Gtf3c1	232	Mipep	302	Pdia5	372	Rpl14	442	Thoc5
23	Ankle2	93	Ddx31	163	Gtpbp1	233	Morf4l1	303	Pds5a	373	Rpl18	443	Tlr2
24	Api5	94	Ddx41	164	Gtpbp10	234	Mov10	304	Pds5b	374	Rpl22l1	444	Tmem199
25	Aqr	95	Ddx46	165	Guf1	235	Mrpl21	305	Pelo	375	Rpl24	445	Tmpo
26	Arhgef2	96	Ddx50	166	Gvin1	236	Mrpl30	306	Pes1	376	Rpl3	446	Toe1
27	Arhgef6	97	Ddx51	167	Hck	237	Mrpl44	307	Pex14	377	Rpl5	447	Tomm40
28	Asrgl1	98	Ddx56	168	Hdac6	238	Mrpl47	308	Phax	378	Rpl8	448	Top2a
29	Atg101	99	Ddx58	169	Heatr1	239	Mrps34	309	Phb2	379	Rps11	449	Top2b
30	Atg2b	100	Dennd4a	170	Hexim1	240	Msh2	310	Phf11	380	Rps9	450	Top3b
31	Atg4b	101	Dgkz	171	Hira	241	Msh6	311	Phip	381	Rrp1	451	Trim56
32	Atp2a2	102	Dhx15	172	Hist1h1c	242	Mta1	312	Pik3ap1	382	Rrp12	452	Trip13
33	Atp6ap1	103	Dhx16	173	Hs2st1	243	Mta2	313	Pik3c2a	383	Rrp8	453	Trrap
34	Atp6ap2	104	Dhx30	174	Hsd17b4	244	Mybbp1a	314	Pik3cd	384	Rtcd1	454	Ttc27
35	Atpaf2	105	Dhx36	175	Hsp90b1	245	Myl12a	315	Pkp2	385	Rtn4	455	Ttc39b
36	Bag6	106	Dhx37	176	Hspa5	246	Naa15	316	Pla2g4a	386	Rtn4ip1	456	Tubgcp2
37	Baz1a	107	Dhx58	177	Hspd1	247	Naa25	317	Plekha2	387	Samd9l	457	Ube2i
38	Baz1b	108	Dhx8	178	Hyou1	248	Nars	318	Plin2	388	Sdad1	458	Ube2o
39	Baz2b	109	Dhx9	179	Ibtk	249	Nat10	319	Plrg1	389	Sdcbp	459	Ubtf
40	BC006779	110	Diablo	180	Icam1	250	Ncapd2	320	Pml	390	Sec24d	460	Uhrf1bp1l
41	Birc6	111	Diexf	181	Idh3a	251	Ncapg	321	Pnn	391	Senp1	461	Upf2
42	Bloc1s2	112	Dimt1	182	Ifi203	252	Ndrg2	322	Pno1	392	Senp3	462	Uqcrq
43	Bms1	113	Dis3	183	Ifi44	253	Ndufa12	323	Pnpt1	393	Setd1a	463	Urb1
44	Bnip1	114	Dkc1	184	Ifih1	254	Ndufa6	324	Pogz	394	Sf3b3	464	Usp19
45	Bptf	115	Dnajc3	185	Igtp	255	Nedd1	325	Polr1a	395	Sin3a	465	Usp25
46	Brix1	116	Dnajc9	186	Ikbkap	256	Nemf	326	Polr1e	396	Skiv2l2	466	Usp48
47	Bst2	117	Dph5	187	Ikbkb	257	Nfx1	327	Polr2a	397	Slc16a1	467	Utp11l
48	Btaf1	118	Dtx3l	188	Il1rn	258	Nipbl	328	Polr2b	398	Slc16a10	468	Utp15
49	Bud31	119	Eef1a1	189	Ilf2	259	Nlrp3	329	Polr3a	399	Slc23a2	469	Utp6
50	Bzw2	120	Eefsec	190	Impad1	260	Nmi	330	Polr3b	400	Slc30a1	470	Wapal
51	C130026I21	121	Eftud2	191	Inpp5b	261	Noc2l	331	Pom121	401	Slc38a2	471	Wdr18
52	C330027C0	122	Eif2b1	192	Ints1	262	Noc3l	332	Pop1	402	Slc3a2	472	Wdr3
53	Ca2	123	Eif2b3	193	Ints3	263	Noc4l	333	Por	403	Slc4a7	473	Wdr36
54	Cad	124	Eif3d	194	Ints4	264	Nol10	334	Ppat	404	Smarca4	474	Wdr43
55	Caprin1	125	Eif3e	195	Ipo13	265	Nom1	335	Ppp2ca	405	Smarca5	475	Wdr46
56	Carm1	126	Eif3l	196	Ipo7	266	Nomo1	336	Ppwd1	406	Smarcad1	476	Wdr47
57	Ccdc134	127	Eif4a1	197	Ipo9	267	Nop56	337	Prkra	407	Smarcal1	477	Wdr75
58	Ccdc47	128	Eif4g2	198	Irf5	268	Npm1	338	Prmt1	408	Smc2	478	Xab2
59	Ccdc50	129	Eml4	199	Irg1	269	Nr3c1	339	Prmt3	409	Smc4	479	Xpc
60	Ccdc72	130	Endod1	200	Irgm2	270	Nsdhl	340	Prmt5	410	Smchd1	480	Xpo5
61	Ccnt1	131	Ensa	201	Isyna1	271	Nt5dc3	341	Prpf31	411	Smpd4	481	Yme1l1
62	Cct4	132	Etnk1	202	Junb	272	Nudcd1	342	Prpf6	412	Smpdl3b	482	Ythdc2
63	Cct8	133	Exosc2	203	Kat7	273	Nudt16l1	343	Prpf8	413	Smu1	483	Zc3h18
64	Cdk5rap2	134	Fam105a	204	Katna1	274	Numa1	344	Ptrh2	414	Snd1	484	Zc3h7a
65	Cebpz	135	FAM120A	205	Kdm1a	275	Nup107	345	Pus1	415	Snrnp200	485	Zc3hav1
66	Cep128	136	Far1	206	Kiaa0020	276	Nup133	346	Pwp2	416	Sp100	486	Zfhx4
67	Chd2	137	Fasn	207	Kiaa0664	277	Nup155	347	Pycr2	417	Sp110	487	Zfr
68	Chd8	138	Fastkd2	208	Kidins220	278	Nup160	348	Rab11fip1	418	Sptlc2	488	Zmat2
69	Cirh1a	139	Fcf1	209	Kif15	279	Nup188	349	Rab31	419	Srp14	489	Znf800
70	Ckap4	140	Fcgr1	210	Kif2a	280	Nup205	350	Rac2	420	Srpk1	490	Znfx1

Human proteins from THP-1 cells

No.	Gene	No.	Gene	No.	Gene	No.	Gene	No.	Gene	No.	Gene
1	ABCB1	71	CSTF1	141	HEATR5B	211	METTL3	281	PSAP	351	SSBIP1
2	ABL2	72	CTNNA1	142	HEBP1	212	MOGS	282	PSMA4	352	STAT1
3	ACBD3	73	CTSL1	143	HGS	213	MORF4L2	283	PSMA7	353	STIP1
4	ACTN4	74	CUTA	144	HIRA	214	MPHOSPH10	284	PSMD13	354	STK38L
5	AHSA1	75	CYLD	145	HIST1H1C	215	MRPL30	285	PSMD8	355	STK39
6	AK2	76	DDX17	146	HK2	216	MRPL39	286	PSMF1	356	SUB1
7	AKAP13	77	DDX39B	147	HMBS	217	MRPL46	287	PTER	357	SUPV3L1
8	ALDOA	78	DDX58	148	HMGB1	218	MRTO4	288	PTK2B	358	SYAP1
9	ALKBH5	79	DDX59	149	HMGB2	219	MSI2	289	PXN	359	SYF2
10	ANKLE2	80	DECR1	150	HMGB3	220	MSR1	290	RAB18	360	SYNCRIP
11	ANKS1A	81	DHRS7	151	HMOX2	221	MSRA	291	RABEP2	361	SYNRG
12	ANP32A	82	DHX38	152	HNRNPA1	222	MTIF3	292	RAD23B	362	TAF4
13	AP2B1	83	DLAT	153	HNRNPA2B1	223	MTMR3	293	RAP1B	363	TALDO1
14	APC	84	DLD	154	HNRNPA3	224	MTPN	294	RBBP5	364	TAP2
15	ARFIP1	85	DLGAP4	155	HNRNPD	225	NAGK	295	RBM10	365	TDO2
16	ARHGAP12	86	DMAP1	156	HNRNPF	226	NANS	296	RBM12	366	TEX264
17	ARHGAP15	87	DNAJA2	157	HPCAL1	227	NAP1L1	297	RBM15	367	TFCP2
18	ARHGAP31	88	DNAJC16	158	HS2ST1	228	NCOA3	298	RBM5	368	TGFB1
19	ARL15	89	DNAJC9	159	HSP90AA1	229	NDST1	299	RBM8A	369	TIMM8A
20	ARL3	90	DRAP1	160	HSPA13	230	NEK6	300	RBMX	370	TLK2
21	ARPC5L	91	DYNC1I2	161	HSPA8	231	NFATC2IP	301	REL	371	TMED8
22	ARSB	92	DYNLL2	162	HSPA9	232	NFU1	302	RELB	372	TMF1
23	ASPSCR1	93	ECHDC1	163	HSPD1	233	NFX1	303	RGC32	373	TMOD3
24	ATM	94	EEA1	164	HSPE1	234	NIT2	304	RNF13	374	TNS3
25	ATP2A3	95	EEF1B2	165	IFI16	235	NKTR	305	RPE	375	TOMM20
26	ATP2C1	96	EFTUD1	166	IFIH1	236	NME2	306	RPL12	376	TOP1
27	ATP5C1	97	EHMT1	167	IFIT1	237	NMI	307	RPL24	377	TRIM13
28	ATP6V1B2	98	EIF2AK2	168	IFIT3	238	NR3C1	308	RPL28	378	TRIM25
29	ATRX	99	EIF2B2	169	IFIT5	239	NUCKS1	309	RPL29	379	TRIM33
30	ATXN10	100	EIF2S1	170	IL1B	240	NUDCD1	310	RPL37	380	TRRAP
31	BCAS2	101	EIF4H	171	IL1RN	241	NUDT5	311	RPLP1	381	TSPYL5
32	BDH1	102	ENO1	172	ILKAP	242	NUFIP2	312	RPS12	382	TTC1
33	BLZF1	103	ENPP4	173	IMPA1	243	NUMB	313	RPS13	383	TTI2
34	C11orf54	104	ENSA	174	IMPDH2	244	NUP155	314	RPS19	384	TUBG1
35	C11orf58	105	EPHX1	175	IPO5	245	NUP188	315	RPS21	385	TXNL1
36	C19orf10	106	ERLEC1	176	IRF2BP2	246	NUP214	316	RPS6	386	UBE2L3
37	C1orf198	107	ERP44	177	IRF2BPL	247	NXT1	317	RPS6KA5	387	UBE3A
38	C1orf31	108	EVI2B	178	ISG20L2	248	NXT2	318	RRP9	388	UFM1
39	CACYBP	109	FAM105A	179	JUNB	249	OAS1	319	RRS1	389	UQCRC1
40	CAMSAP1	110	FAM105B	180	KIAA1468	250	OAS2	320	S100A8	390	USP11
41	CAPZA1	111	FAM136A	181	KIAA1598	251	OPTN	321	S100A9	391	USP40
42	CARD6	112	FAM82B	182	LACTB2	252	OSBPL8	322	SACM1L	392	UTP14A
43	CARHSP1	113	FBXO6	183	LAMP2	253	OSTF1	323	SARNP	393	UTP20
44	CASP4	114	FCF1	184	LASP1	254	P4HB	324	SEC22B	394	VAPA
45	CBX3	115	FNBP4	185	LCLAT1	255	PAG1	325	SEC24C	395	VPS25
46	CBX5	116	FOSL2	186	LCP2	256	PAICS	326	SEC62	396	VPS29
47	CCDC149	117	FOXK2	187	LEPRE1	257	PARK7	327	SEMA7A	397	WARS2
48	CCDC50	118	FUBP3	188	LGALS1	258	PARP14	328	SEPT6	398	WDR11
49	CCDC56	119	FUS	189	LGALS3	259	PARP9	329	SFXN1	399	WDR75
50	CCDC58	120	G3BP1	190	LIMK1	260	PDIA3	330	SGK223	400	XIAP
51	CCL20	121	G3BP2	191	LMNA	261	PDSS2	331	SIPA1L1	401	XIRP1
52	CCPG1	122	GABPA	192	LPL	262	PEA15	332	SLAIN2	402	YBX1
53	CCT5	123	GAPDH	193	LRCH1	263	PELO	333	SLC12A6	403	YWHAE
54	CCZ1B	124	GBP5	194	LRP12	264	PFDN5	334	SLC20A1	404	ZBTB7A
55	CD109	125	GCC2	195	LRPAP1	265	PGK1	335	SLC25A1	405	ZCCHC6
56	CD276	126	GCLM	196	LRRC8D	266	PHC2	336	SLTM	406	ZCCHC8
57	CD38	127	GCSH	197	LRRFIP1	267	PHF6	337	SMYD5	407	ZDHHC5
58	CD40	128	GGCT	198	LRRFIP2	268	PHLDA1	338	SNRPC	408	ZFP106
59	CDC37	129	GLIPR2	199	LYRM7	269	PIKFYVE	339	SNX17	409	ZFP36L1
60	CDK12	130	GLO1	200	MALT1	270	PIN1	340	SNX8	410	ZFP91
61	CEBPB	131	GLOD4	201	MAN1A1	271	PLEKHO2	341	SOD2	411	ZFYVE16
62	CHTOP	132	GTF2F1	202	MAP3K2	272	PLSCR1	342	SP110	412	ZMYM2
63	CLASP2	133	GTF2F2	203	MAPKAP1	273	PNPO	343	SPAG1	413	ZNF281
64	CLPX	134	GTF3C4	204	MARK2	274	PNPT1	344	SPAG7	414	ZNF592
65	CORO1A	135	H1F0	205	MARK3	275	PPIA	345	SPI1		
66	CPSF2	136	HAT1	206	MAZ	276	PPIG	346	SPP1		
67	CRIP1	137	HCFC1	207	MCM3	277	PPM1B	347	SRRM2		
68	CRK	138	HCLS1	208	MCM4	278	PPP1R9B	348	SRSF1		
69	CRNKL1	139	HDGF	209	MCM5	279	PRDX1	349	SRSF2		
70	CRYBB1	140	HDHD1	210	MCM7	280	PRDX5	350	SRSF6		

Supplementary Table 4: List of genes evaluated in single cell analysis

No.	Gene	No.	Gene	No.	Gene	No.	Gene
1	ABCA1	31	GALANT2	61	Nfe2L2	91	VPS4
2	ABCD1	32	GJA1	62	NFKB		
3	ABCG1	33	HOMX1	63	NOS2		
4	ACTA2	34	HPRT1	64	NUDCD1		
5	ADM	35	IFNGR1	65	OAS1		
6	ARG1	36	IL10	66	OLR1		
7	ATF1	37	IL12B	67	PARP1		
8	CCL2	38	IL1b	68	PARP14		
9	CCL22	39	IL4R	69	PARP2		
10	CCL5	40	IL6	70	PARP9		
11	CCND1	41	IL8	71	PDCD4		
12	CD14	42	IRF1	72	PGC1a		
13	CD200R	43	IRF2	73	PPARa		
14	CD36	44	IRF3	74	PPARg		
15	CD80	45	IRF5	75	S100A9		
16	CD86	46	IRF8	76	SNX1		
17	COL1A1	47	IRF9	77	SNX2		
18	CPT1a	48	IRG1	78	SORT1		
19	CPT1b	49	JAK1	79	Sp110		
20	CTSK	50	JAK2	80	SRA		
21	CXCL10	51	JAK3	81	STAT1		
22	CXCL11	52	KLF4	82	STAT2		
23	CXCL6	53	LAMP2	83	STAT3		
24	CXCL6	54	LDLR	84	STAT6		
25	DTX3L	55	MMP1	85	TAGLN		
26	EGR1	56	MMP12	86	TCF4		
27	EGR2	57	MMP8	87	TLR2		
28	F3	58	MMP9	88	TLR4		
29	FCGRT	59	MRC1	89	TNF		
30	GADD45a	60	MYC	90	TRIB1		
